# Identification of physicochemical properties that modulate nanoparticle aggregation in blood

**DOI:** 10.3762/bjnano.11.44

**Published:** 2020-04-03

**Authors:** Ludovica Soddu, Duong N Trinh, Eimear Dunne, Dermot Kenny, Giorgia Bernardini, Ida Kokalari, Arianna Marucco, Marco P Monopoli, Ivana Fenoglio

**Affiliations:** 1 Department of Chemistry, University of Torino, 10125 Torino, Italy; 2 Molecular and Cellular Therapeutics, Royal College of Surgeons in Ireland (RCSI), 123 St Stephen Green, Dublin 2, Ireland; 3 Department of Chemistry, Royal College of Surgeons in Ireland (RCSI), 123 St Stephen Green, Dublin 2, Ireland

**Keywords:** aggregation, nanoparticles, platelet aggregation, size, surface chemistry

## Abstract

Inorganic materials are receiving significant interest in medicine given their usefulness for therapeutic applications such as targeted drug delivery, active pharmaceutical carriers and medical imaging. However, poor knowledge of the side effects related to their use is an obstacle to clinical translation. For the development of molecular drugs, the concept of safe-by-design has become an efficient pharmaceutical strategy with the aim of reducing costs, which can also accelerate the translation into the market. In the case of materials, the application these approaches is hampered by poor knowledge of how the physical and chemical properties of the material trigger the biological response. Hemocompatibility is a crucial aspect to take into consideration for those materials that are intended for medical applications. The formation of nanoparticle agglomerates can cause severe side effects that may induce occlusion of blood vessels and thrombotic events. Additionally, nanoparticles can interfere with the coagulation cascade causing both pro- and anti-coagulant properties. There is contrasting evidence on how the physicochemical properties of the material modulate these effects. In this work, we developed two sets of tailored carbon and silica nanoparticles with three different diameters in the 100–500 nm range with the purpose of investigating the role of surface curvature and chemistry on platelet aggregation, activation and adhesion. Substantial differences were found in the composition of the protein corona depending on the chemical nature of the nanoparticles, while the surface curvature was found to play a minor role. On the other hand, large carbon nanoparticles (but not small carbon nanoparticles or silica nanoparticles) have a clear tendency to form aggregates both in plasma and blood. This effect was observed both in the presence or absence of platelets and was independent of platelet activation. Overall, the results presented herein suggest the existence of independent modes of action that are differently affected by the physicochemical properties of the materials, potentially leading to vessel occlusion and/or formation of thrombi in vivo.

## Introduction

Nanomedicine is one of the most exciting fields of research in the branch of nanotechnology as it has the potential to generate practical and effective solutions to tackle chronic diseases and to solve unmet clinical challenges. However, a tremendous gap exists between the number of numerous formulation types synthesised in research laboratories and those approved for clinics [1], mainly due to the lack of understanding on the nanoparticles (NPs) behaviour in complex media that can affect their efficacy and their biocompatibility [2].

Safe-by-design (SbD) approaches have great potential in accelerating the entry of medicines into the market [3] with the aim of reducing the preclinical research time and the associated costs for production. A deep knowledge of the processes leading to the adverse effects and of the physicochemical properties governing such processes are required to build structure–activity relationships (SARs) that in turn enable the SbD approaches. To the latter aspect, knowledge can only be derived by substantial screening of libraries of nanomaterials with well defined synthetic properties.

The understanding of the processes occurring in the bloodstream is particularly relevant not only for nanoformulations administered by intravenous injection, but also for any material that is introduced into the body by other routes as they have the potential to cross biological barriers and subsequently enter into the bloodstream. Previous studies have shown that specific NPs have been able to bind to biomolecules from the coagulation system and induce haemorrhage or thrombosis [4]. The depletion of soluble coagulation factors (e.g., fibrinogen, XII factor) may occur following adsorption of the factors at the NP surface. On the other hand, the activation of some factors by surface-driven exposure of cryptic domains following adsorption was reported in some studies [5–6]. Other studies have reported the NPs ability to damage or activate platelets, endothelial cells or monocytes [4].

Some physicochemical properties, including the surface charge and the particle size, were found to be critical in influencing the nanomaterial's ability to induce adverse effects [7]. However, although such properties were shown to clearly affect the pro-/anticoagulant activity of NPs, the direction of the effect varies depending on the tested material, and it is currently not clear which properties lead to the activation of the coagulation. For example, positive charged dendritic NPs were found to be more thrombogenic than negatively charged ones while positive and negative charged polystyrene NPs both induce platelet activation [7–8].

Carbon and silica nanomaterials are among the most studied inorganic materials for medical applications due to their promising properties. However, some studies have shown that they are both capable of inducing the formation of thrombi, and the relevant mechanisms of action are still under debate [9]. In fact, single-walled (SWCNTs) and multiwalled carbon nanotubes (MWCNTs) can induce platelet activation by inducing depletion of intracellular Ca^2+^ [10–11], an effect that was hypothesised to be caused by the interaction of CNTs with plasma and dense tubular system membranes likely related to the fibrous shape [12]. On the other hand, contrasting data have been reported on the potential of isometric carbon nanoparticles (CNPs) like carbon black, fullerenes and diesel exhaust particles to induce platelet activation and NP aggregation [10–11,13]. Systemic administration of carbon black in mice resulted in fibrinogen and platelet deposition in post-capillary venules in the liver and heart, suggesting the role of this protein in nanoparticle-mediated platelet aggregation [14–15].

Silica nanoparticles (SNPs) of different sizes were found to activate glycoprotein IIb/IIIa and to induce the expression of P-selectin in platelets [16]. Additionally, SNPs were found to induce pre-thrombotic states through surface-driven activation of the coagulation factor XII [17–18]. Finally, SNPs are known to induce oxidative stress in several cell lines including endothelial cells [19] and leucocytes [20–21], a process that in vivo may indirectly induce platelet aggregation.

The interference with the coagulation system is not the only possible mechanism that may induce vascular occlusion, as the NP have a strong tendency to agglomerate also in water. The degree of the agglomeration is controlled by the size, shape and surface chemistry of the particles. Strong repulsive electrostatic charges and steric hindrance may stabilize the NPs and prevent agglomeration. In the bloodstream, agglomeration is related to the formation of a biocorona that modifies the electrostatic and steric repulsion among particles [22]. Finally, protein–protein interaction may lead to bridging among particles, thus promoting agglomeration [23].

In the present study, a set of six silica and carbon NPs of known size and morphology was used to evaluate the effect of the size and surface properties on the protein corona composition, platelet activation and aggregation.

## Materials and Methods

### Reagents

Sodium polyacrylate, ᴅ-(+)-glucose, thionine acetate salt, phosphate buffered saline powder, EDTA, glutathione reduced and 5,5′-dithiobis(2-nitrobenzoic acid), tetraethyl orthosilicate (TEOS), phosphate buffered saline (PBS) tablets were obtained from Sigma-Aldrich (Germany). 5,5-dimethyl-1-pyrroline *N*-oxide (DMPO) was obtained from Cayman Chemicals (USA). Ultrapure water was obtained from a Milli-Q Plus system (Millipore, Bedford, MA, USA). All other chemicals and solvents used were at least of analytical grade.

### Synthesis of carbon nanoparticles

Carbon nanoparticles (CNPs) were produced starting from glucose using a one-step hydrothermal process as previously described by Kokalari et al. 2019 [24]. Briefly, glucose was dissolved in 50 mL of ultrapure water followed by the addition of 15 mg of sodium polyacrilate. The solution was introduced in a pressure reactor system (Büchi AG) and heated at 190 °C for 3 or 8 h. The parameters used during the synthesis are described in detail in Table 1. The CNPs were then purified with ultrapure water either by centrifugation for large carbon nanoparticles (CNP-L) or by tangential flow ultrafiltration (Vivaflow 50R, MW 30 kDa) for the medium and small carbon nanoparticles (CNP-M and CNP-S, respectively).

**Table 1:** Synthesis parameters used for the CNPs.

	Glucose (g)	Surfactant (mg)	Time (h)	Temperature (°C)

CNP-S	2	15	3	190
CNP-M	2	15	8	190
CNP-L	5	15	8	190

### Synthesis of silica nanoparticles

Silica nanoparticles (SNPs) were prepared by hydrolysis and condensation of TEOS in the presence of ammonia as a catalyst following the Stöber process [25]. Briefly, a defined amount of TEOS was added to a solution containing ethanol, ammonia (33%) and ultrapure water under magnetic stirring and at room temperature for 30–40 min. The ratio of the reagents was modified in order to control the NPs size (Table 2). The NP suspension was centrifuged at 11,000 rcf for 15 min and the particles re-suspended in ethanol once, centrifuged, and re-suspended in ultrapure water. The procedure was repeated three times. The purified NPs were suspended in ultrapure water and stored at 4 °C until use.

**Table 2:** Synthesis parameters for SNPs.

	TEOS (mL)	Ethanol (mL)	NH_3_ (mL)	H_2_O (mL)	Time (min)

SNP-S	0.76	20	0.85	0.83	40
SNP-M	0.76	20	1.70	1.07	40
SNP-L	0.33	44	18	–	30

### Scanning electron microscopy (SEM)

The NPs morphology was characterised using scanning electron microscopy (SEM), using a Zeiss Evo 50XVP (Assing) instrument. CNPs and SNPs suspensions were diluted up to 0.05 mg/mL in ultrapure water. A volume of 20 μL of the diluted suspensions was mounted on aluminium stubs using double-sided adhesive carbon tape and silicon wafers. The samples were dried overnight at room temperature. In the case of SNPs, the samples were sputter-coated with a thick gold film (≈17 nm) under argon atmosphere to improve secondary electron emission during SEM imaging. The NPs morphology was observed at an acceleration voltage of 20 kV.

### Dynamic light scattering (DLS)

The mean diameter and polydispersity index (PDI) of the NPs were obtained using a Zetasizer (Nano ZS Malvern Instruments, UK) instrument based on the dynamic light scattering (DLS) technique. The measurements were performed on purified NPs by analysing 0.5 mL of the suspension in ultrapure water, placed in a square polystyrene cuvette, at 25 °C. PBS 0.01 M, pH 7.4, Sigma-Aldrich, was used as the diluent in the case of the evaluation of the size after the protein corona formation.

### Nanoparticle tracking analysis (NTA)

An analysis of the size distribution and concentration of CNPs and SNPs were performed by NTA using a Nanosight NS300 (Malvern, UK) instrument equipped with a blue laser (488 nm) and a quartz chamber for sample injection, equipped with an O-ring top plate. For the NP/hard protein corona complexes, the samples were diluted in PBS (0.01 M, pH 7.4). The dilution factor was chosen in order to obtain 30 particles per frame, as suggested by the manufacturer’s recommendations. The measurement duration was set at 60 s.

### Zeta potential

Zeta-potential measurements were performed based on the electrophoretic light scattering (ELS) technique, using a Zetasizer (Nano ZS Malvern Instruments, UK) instrument, as a function of the pH in the range from 2–9. The NP suspensions were diluted in ultrapure water at a final concentration of 0.5 mg/mL. The pH of the suspensions was adjusted by adding diluted NaOH or HCl solutions and the samples were introduced into disposable folded capillary cuvettes (Malvern Panalytical).

### Protein corona characterization

#### Access to blood plasma for the corona study

The blood plasma used for the corona studies was obtained from the Irish Blood Transfusion service (IBTS) St Vincent’s Hospital, Dublin. The plasma, derived from eight different donors, was polled, aliquoted and stored −80 °C until use. The use of this biological fluid for corona studies is covered by the RCSI REC 1246b.

#### Methods

On the day of the experiment, an aliquot of blood plasma was thawed and allowed to reach room temperature. Once thawed, the sample was centrifuged for 3 min at 16,000 rcf in order to pellet any aggregated proteins. The supernatant was then used for the incubation step while the pellet was discarded.

Blood plasma was diluted in PBS in order to obtain solutions with increasing protein. CNPs and SNPs were then added to the solution and were incubated for 1 h at 37 °C under agitation (150 rpm). Sample normalisation was carrred out throughout the experiments to ensure total surface area of 1.0 × 10^−2^ m^2^ per incubation step.

After the NPs incubation with human plasma, the nanoparticle–protein corona complexes were pelleted by centrifugation and re-suspended in PBS three times in order to remove the loosely binding coronas as previously described [26]. After the last washing step, the pelleted samples were suspended in 20 μL PBS and 10 μL of 3X Blue Loading Buffer Reagents (New England biolabs) that contained DTT in a ratio of 1:10 following the manufacturer instructions. The samples were sonicated for 5 min in an ultrasonic bath and then heated for 5 min at 95 °C to complete the protein denaturation.

The protein corona was resolved in a 4% stacking gel / 12% acrylamide and the electrophoretic analysis was conducted at 130 V as previously described [26]. After the electrophoretic separation, the gels were stained in Imperial Protein stain (Thermo Scientific) for 1 h and distained overnight (in ultrapure water). The densitometry analysis was performed using the software ImageJ (NIH).

#### Mass spectrometry analysis

The samples were run on a sodium dodecyl sulfate–polyacrylamide gel electrophoresis (SDS-PAGE) instrument for 10 min before the protein bands were excised from the gel in order to allow the whole corona proteins to migrate into a single gel band. The proteins in the gel pieces were reduced with dithiothreitol, alkylated with iodoacetamide and digested with trypsin (Promega Corporation) overnight at 37 °C. The peptides were then extracted from the gel matrix and prepared for MS analysis by using Pierce C18 Tips (Thermo Fisher) following the manufacturer's procedure.

The peptide samples were analysed on a quadrupole Orbitrap (Q-Exactive, Thermo Scientific) mass spectrometer equipped with a reversed-phase NanoLC UltiMate 3000 HPLC system (Thermo Scientific). The samples were loaded onto C18 reversed phase columns (10 cm length, 75 µm inner diameter) and eluted with a linear gradient from 2 to 27% acetonitrile containing 0.5% acetic acid in 58 min at a flow rate of 250 nL/min. The injection volume was 5 μL. The mass spectrometer was operated in data dependent mode, automatically switching between MS and MS2 acquisition. Survey full scan MS spectra (*m*/*z* 300–1600) were acquired in the Orbitrap with a resolution of 70,000. MS2 spectra had a resolution of 17,500. The twelve most intense ions were sequentially isolated and fragmented by higher-energy C-trap dissociation.

MS raw files were processed with MaxQuant software (version 1.6.2). The peak lists were searched against the human FASTA database. The search included the modifications of cysteine carbamidomethylation, methionine oxidation and protein N-terminal acetylation. A maximum of two missed trypsin cleavages were allowed in the database search. The false discovery rate for both peptides and proteins was set at 1%. After that, the ProteinGroup file from Maxquant was processed, filtered and analysed with Perseus software to generate the top abundance table, hierarchical clustering graph and numeric Venn diagrams.

### Preparation of blood and platelet-rich plasma (PRP)

#### Access to human blood for the platelet aggregation study

Blood collection for this study was approved by the Royal College of Surgeons in Ireland and Beaumont Hospital ethics committees REC1415. Written informed consent was obtained from all donors prior to phlebotomy. All blood samples were taken in accordance with the declaration of Helsinki.

#### Study participants

25 healthy donors were recruited for this study. All donors had no previous history of any major disease and were free from any medication such as statins, antihypertensive medication, antiplatelet agents such as aspirin, or anti-inflammatory medications such ibuprofen, for at least 12 days prior to blood draw.

#### Preparation of blood and platelet-rich plasma (PRP)

Venous blood was drawn from the antecubital vein using a 19-gauge butterfly needle connected to a sterile polypropylene syringe. Blood was drawn into 3.2% (w/v) trisodium citrate anticoagulant (1:9 volume of citrate to blood, final citrate concentration of 0.32%). Blood samples were kept at room temperature with gentle rocking and used within 1 h of phlebotomy. Whole blood cell counts were recorded for each donor, using a Sysmex-KX21N haematology analyser (Kobe, Japan). Blood samples were centrifuged at 170*g* for 10 min to obtain platelet-rich plasma (PRP) (Centrifuge 5417R, BIOTOOLS, CA).

### Light transmission aggregometry (LTA)

Platelet aggregation was monitored by light transmission aggregometry (LTA) in a Chronolog-490D aggregometer (CHRONO-LOG^®^ Corporation, Havertown, PA). Adenosine diphosphate (ADP) and collagen were used as activators of platelet aggregation.

The NPs were first suspended in phosphate buffered saline (PBS) and then they were added to 250 µL PRP and incubated for 1 min. The concentration of NPs to use during the incubation step was calculated on the base of mean diameter (DLS) and nanoparticle concentration number (NTA) to have a total of 8.6 × 10^−4^ m^2^/mL of exposed surface area, equal to 1 mg/mL for CNP-S. In the case of CNP-L the concentration was lower to avoid interference (7.25 × 10^−5^ m^2^/mL). 2.5 μL of 10 μM ADP or 12.5 μL of 10 μM collagen was then added and aggregation was monitored for 5 min with the suspension continuously stirred. The optical density was also measured for NPs suspended in PRP in the absence of agonists for up to 40 min of incubation. PRP without NPs was used as the control. The data are expressed as the mean of three independent experiments.

### Flow cytometry fluorescence-activated cell sorting (FACS)

Flow cytometry fluorescence-activated cell sorting (FACS) was used to evaluate platelet activation. The NPs were suspended in PRP at the same concentration used for LTA. 2.5 µL of the fluorophore-conjugated antibody (CD62P) (1.5 µg/mL) (Becton Dickinson, Oxford, UK), which binds P-selectin, and 87.5 µL of PBS were added to 10 µL of the suspension and incubated for 10 min. The reactions were stopped by the addition of PBS to a final volume of 1 mL. The mean fluorescence intensity (MFI) was read on a Beckman Coulter Cytomics FC500 flow cytometer. The experiments were repeated by adding 5 µL of ADP (10 µM). The data are expressed as the mean of three independent experiments.

### Dynamic platelet function assay (DPFA)

The DPFA is a well-characterised real-time assay of platelet interaction with von Willebrand factor (VWF) under conditions of arterial shear [27–29].

The initial phases of platelet aggregation were assayed using the DPFA as previously described [25–26]. Briefly, custom parallel plate perfusion chambers were coated overnight with 100 μg/mL VWF, washed with phosphate-buffered saline and blocked with 1% bovine serum albumin for 1 h prior to use. Whole blood was labelled with 1 μM DiOC_6_ (Sigma-Aldrich, Ireland) for 5 min at 37 °C prior to perfusion through the chamber at an arterial rate of shear (1500 s^−1^). Platelet translocation behaviour was recorded using real-time video microscopy at a rate of 19 frames per second. Image stacks were analysed by a custom-designed and validated software package [27]. The assay measurements obtained from this analysis include the number of platelets that interacted with the VWF surface (platelet tracks), the number of platelets that translocate over VWF (translocating platelets), the average speed at which platelet translocation occurred (platelet translocation speed), the distance a platelet translocated along the VWF surface (platelet translocation distance), the number of platelets that stably adhered to the VWF-coated surface (static platelets), and the percent surface coverage on the final frame (percentage of platelet surface coverage). For this study we only considered the platelet adhesion parameters.

## Results

### Synthesis and characterisation of the libraries of silica and carbon nanoparticles

In this study, we synthesised two matching sets of SNPs and CNPs that had a similar hydrodynamic diameter. The mean hydrodynamic diameter based on DLS and NTA confirmed a similar size distribution between the two materials (Table 3). The low polydispersity index (PDI) indicates high colloidal stability and narrow size range distribution.

**Table 3:** Mean hydrodynamic diameter, PDI and standard deviation of each sample measured after purification obtained with DLS compared with the mean of hydrodynamic diameter and standard deviation obtained using NTA.

	DLS			NTA	

	Hydrodynamic diameter (nm)	Standard dev.	PDI	Hydrodynamic diameter (nm)	Standard dev.

SNP-S	114.1	±0.351	0.081	115.0	±1.52
SNP-M	235.1	±4.754	0.012	217.3	±4.16
SNP-L	488.1	±5.387	0.031	333.3	±14.5
CNP-S	179.5	±3.482	0.074	128.3	±2.52
CNP-M	259.7	±2.193	0.010	232.0	±6.08
CNP-L	485.2	±2.452	0.123	349.0	±4.36

The hydrodynamic diameter of the samples measured by DLS was similar to that measured by NTA, with the exception of the large samples, where the detected diameter with the latter technique was lower than the ones detected with the DLS. In fact, NTA detected four populations of particles of different size (Supporting Information File 1, Figure S1), while in DLS they appeared as a unique polydisperse population. This data explains also the higher PDI values obtained for the large nanoparticle samples comparing to the small ones.

**Figure 1:**
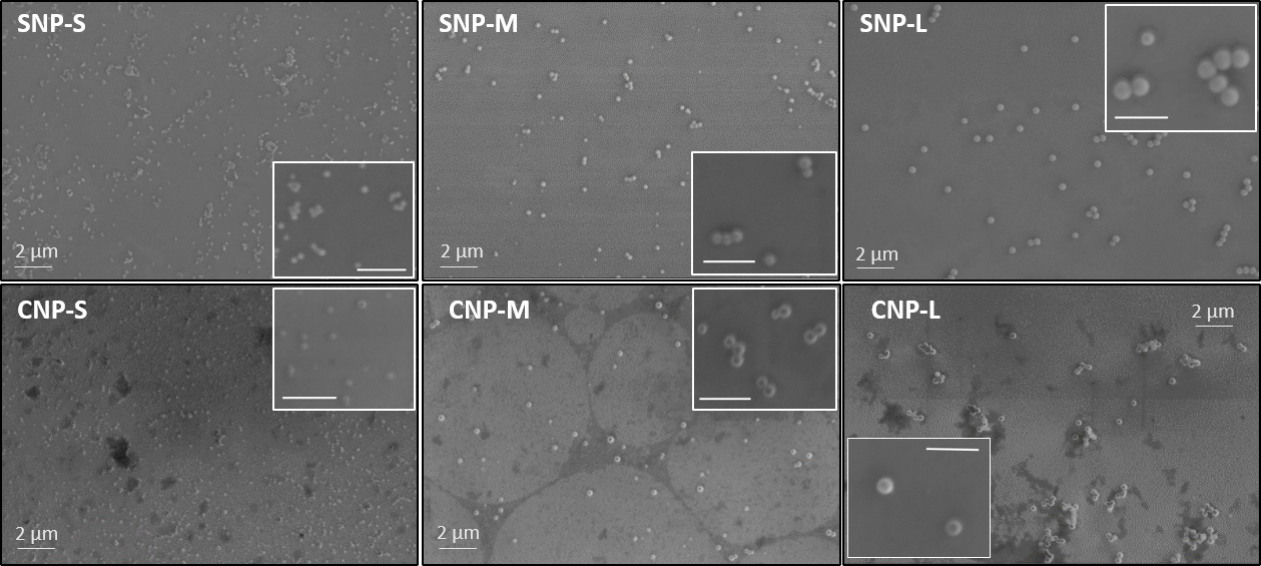
Representative SEM micrographs of silica and carbon nanoparticles. The scale bar in each inset is 1 μm.

SEM analysis confirmed that all particles appear spherical with a uniform size, confirming the DLS analysis (Figure 1).

**Figure 2:**
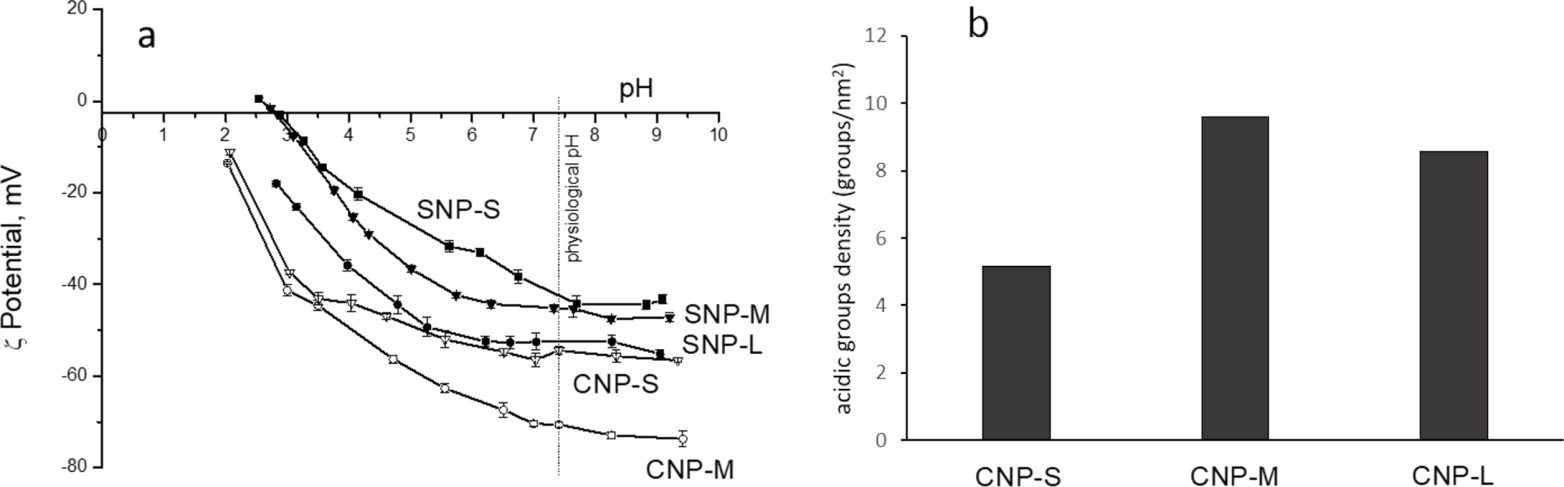
a) Zeta potential versus pH curves for carbon nanoparticles (CNP-M and CNP-S) and silica nanoparticles (SNP-L, SNP-M and SNP-S) suspended in water. b) Density of acid groups exposed at the surface of carbon nanoparticles.

The zeta potential of the samples was measured by electrophoretic light scattering (ELS) in the pH range from 2 to 9 (Figure 2a). As expected, both SNPs and CNPs exhibited a negative zeta potential across the whole pH range. It gradually increased with the increase of the pH of the suspension although it never reached positive values, indicating the presence of weakly acidic groups at the surface. In the case of CNPs, acidic carboxylic or phenolic groups formed during the synthesis are expected, while the presence of surface hydroxyl groups are expected for SNPs. At physiological pH (7.4), all particles exhibit a zeta potential in the range of −40 to −70 mV. CNPs exhibit a zeta potential more negative than the corresponding SNPs of the same size range. Note that the zeta potential curve of CNP-L is not reported since this sample rapidly agglomerates by lowering the pH value, making the measurement unfeasible.

The presence of acidic groups at the surface of CNPs was quantified by titration using the dye thionine acetate [30]. The density values of acidic groups for the CNPs are shown in Figure 2b. The three samples slightly differ in terms of the density of acidic surface groups, with the small NPs having the lowest density in agreement with the observed less negative zeta potential value. The density of the acidic hydroxyl groups for SNPs was not determined here since the value is available in literature [31–33].

### Physicochemical and proteomics characterisation of the nanoparticle/hard corona

**Figure 3:**
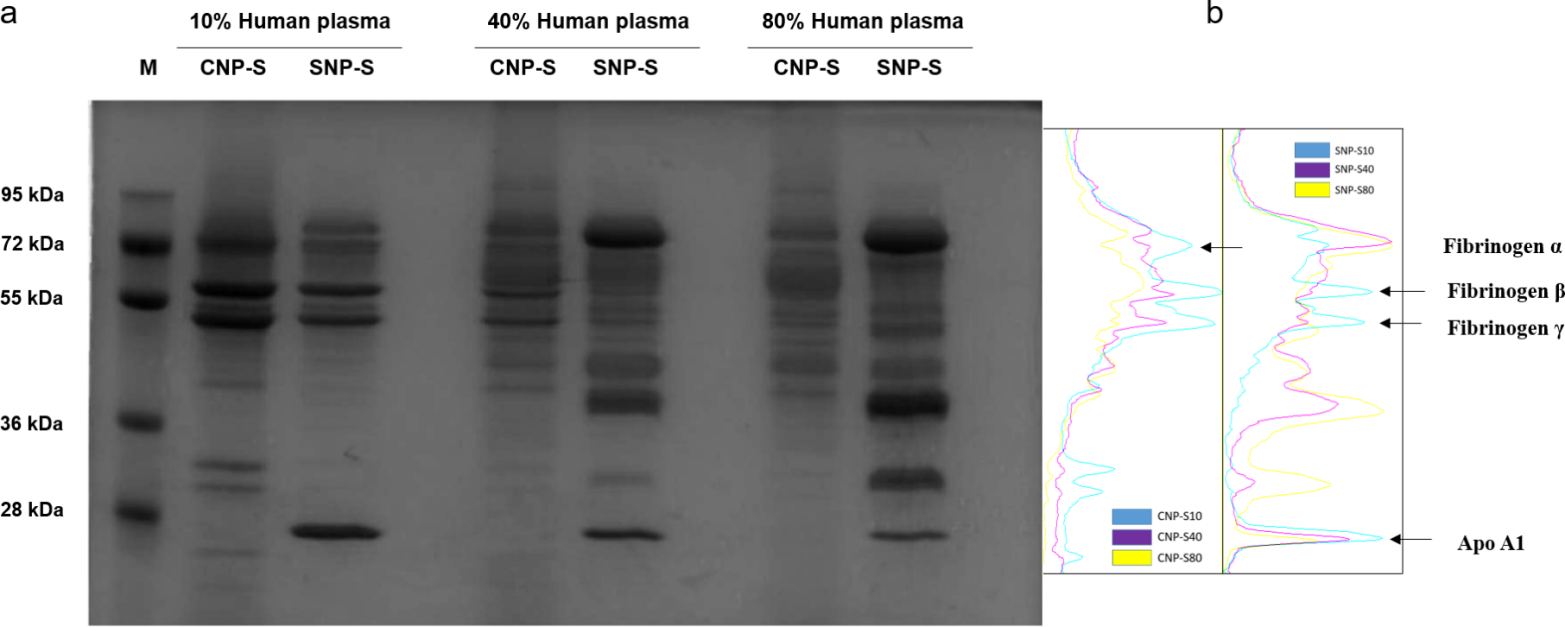
a) SDS-PAGE gel of hard protein corona formed after 1 h of incubation in human plasma. b) Densitometry of the gel bands corresponding to fibrinogen and apolipoprotein A1.

We then evaluated how the NP physicochemical properties would affect the biomolecular corona formation. For this purpose, we exposed the same surface area of silica and carbon NPs of three different sizes to an increasing concentration of human plasma, from 10% to 80%, to mimic the in vitro and in vivo conditions, respectively. In Figure 3 the we show the SDS-PAGE gels for the small silica and carbon nanoparticles. The corona composition between the two materials has some similarities when incubated at 10% plasma; however, it becomes highly specific to the NP surface properties at higher concentrations as confirmed by the significant difference in the corona composition.

In particular, at 10% of plasma, both NPs preferentially adsorb three gel bands of 72, 60 and 50 kDa, later identified as fibrinogen alpha, beta and gamma chain, respectively. Significant differences were also observed at a lower molecular weight where a gel band of 25 kDa was detected in the silica corona only, while in the carbon NP corona, 3 gel bands formed in the region of 36–30 kDa in addition to a less pronounced band of MW lower than 28 kDa. At higher plasma concentration, the corona composition of the SNP changed significantly where the fibrinogen gel bands were displaced by three predominant bands of 90 kDa and a duplet of 50 kDa, later identified as histidine-rich glycoprotein. These findings are in agreement with a previous study where a similar effect was detected for 200 nm SNPs [26].

Small differences in the protein corona composition of NPs of different sizes were found (Supporting Information File 1, Figure S2), suggesting that the surface curvature plays a minor role. This is only apparently in contrast with a previous study showing more significant differences in the protein corona for NPs of different size [34]. In fact, the size range considered here is even larger than in the referenced study.

**Figure 4:**
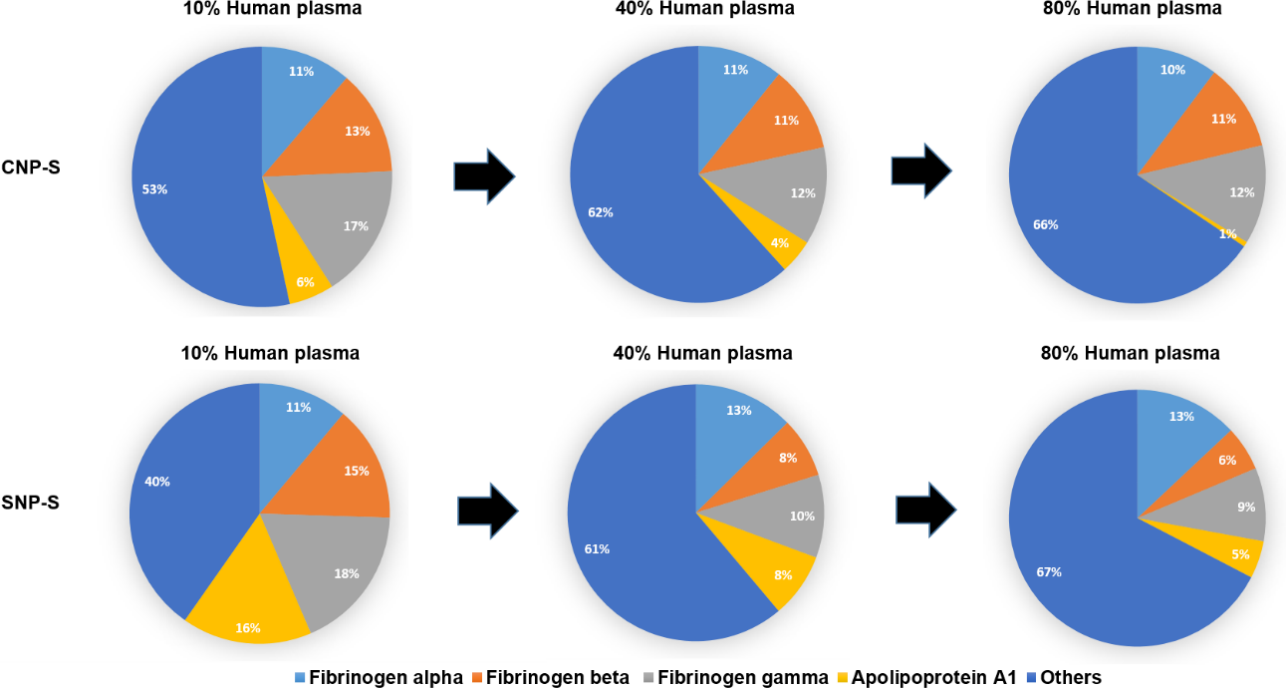
Abundance of fibrinogen and apolipoprotein A1 in silica and carbon nanoparticle corona at different plasma concentrations.

Label-free mass spectrometry analysis was used to obtain the semiquantitative protein abundance of the corona across three different plasma concentrations. Notably, the percent of fibrinogen varies greatly across all conditions while remaining an abundant corona binder protein (Figure 4).

A total of 118 proteins were found in the biomolecular corona of small carbon and silica NPs after incubation with human plasma at 10, 40 and 80%. Table 4 contains the top 20 proteins detected in each condition by MS.

**Figure 5:**
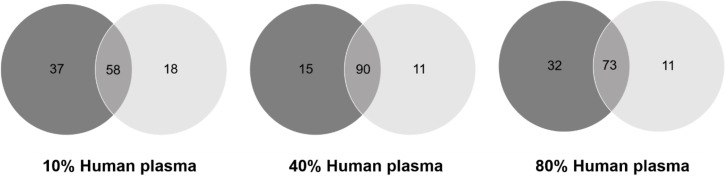
Venn diagrams showing the number of proteins shared by small silica (black) and carbon nanoparticle (grey) protein corona formed at different plasma concentrations.

**Figure 6:**
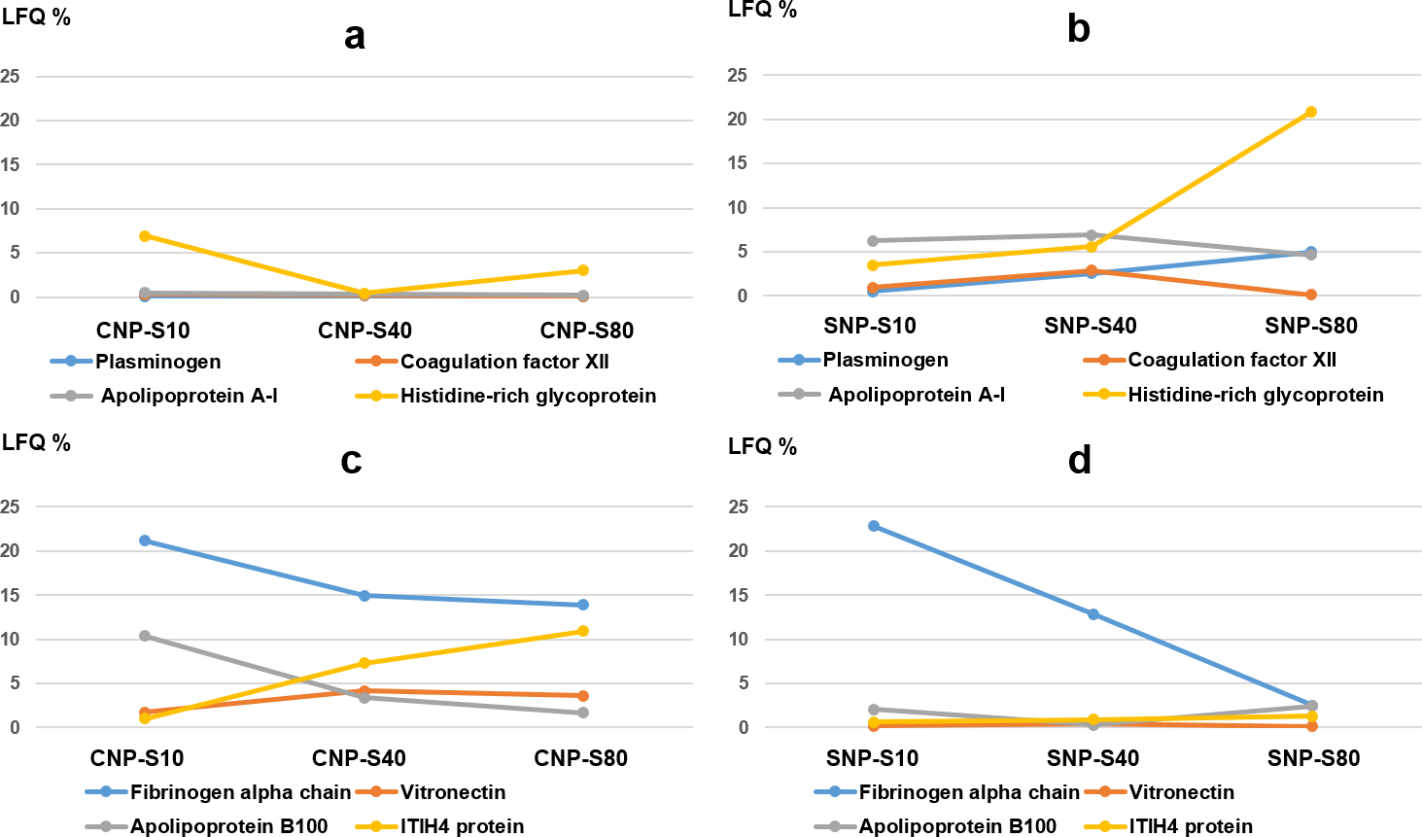
Relative concentration of proteins on small silica (SNP-S) and carbon (CNP-S) nanoparticle coronas at plasma concentrations of 10, 40 and 80%. a–d): semiquantitative protein abundance – comparison between CNP-S and SNP-S coronas. The percentages were calculated based on the total LFQ intensity in each sample.

Venn diagrams (Figure 5) highlight that the majority of the proteins were detected both in the SNP-S and CNP-S at higher plasma concentrations, while a minor overlap occurred at 10%. However, a pronounced difference was observed when we compared the protein abundance by means of the label-free quantification (LFQ) across all conditions (Table 4 and Figure 6).

**Table 4:** Top 20 most abundant proteins in small silica (SNP-S) and carbon (CNP-S) nanoparticle hard corona samples at three different plasma concentrations (10, 40, 80%) based on the LFQ intensity.

Order of abundance	SNP-S			CNP-S		

	10% human plasma	40% human plasma	80% human plasma	10% human plasma	40% human plasma	80% human plasma

1	fibrinogen gamma chain	kininogen-1	kininogen-1	fibrinogen beta chain	fibrinogen beta chain	kininogen-1
2	fibrinogen alpha chain	fibrinogen alpha chain	histidine-rich glycoprotein	fibrinogen alpha chain	fibrinogen gamma chain	fibrinogen alpha chain
3	fibrinogen beta chain	fibrinogen beta chain	kallikrein B	fibrinogen gamma chain	fibrinogen alpha chain	ITIH4 protein
4	apolipoprotein A-I	fibrinogen gamma chain	coagulation factor XI	apolipoprotein B-100	kininogen-1	fibrinogen beta chain
5	kininogen-1	apolipoprotein A-I	plasminogen	histidine-rich glycoprotein	ITIH4 protein	fibrinogen gamma chain
6	apolipoprotein E	histidine-rich glycoprotein	apolipoprotein A-I	kininogen-1	vitronectin	coagulation factor XI
7	histidine-rich glycoprotein	kallikrein B	plasma protease C1 inhibitor	vitronectin	apolipoprotein B-100	vitronectin
8	apolipoprotein B-100	coagulation factor XI	fibrinogen alpha chain	complement C1q	plasma kallikrein	kallikrein B
9	kallikrein B	apolipoprotein E	apolipoprotein B-100	complement component 4B	apolipoprotein E	histidine-rich glycoprotein
10	plasma protease C1 inhibitor	coagulation factor XII	fibrinogen beta chain	complement factor H	complement component 4B	apolipoprotein B-100
11	selenoprotein P	selenoprotein P	fibrinogen gamma chain	apolipoprotein E	coagulation factor XI	apolipoprotein E
12	coagulation factor XII	plasminogen	serum albumin	ITIH4 protein	serum albumin	serum albumin
13	ITIH4 protein	plasma protease C1 inhibitor	ITIH4 protein	serum albumin	complement factor H	complement C3
14	serum albumin	serum albumin	selenoprotein P	complement component 1	complement component 1	Ig gamma-3 chain C region
15	complement C3	ITIH4 protein	apolipoprotein E	complement C3	complement C3	isoform C of Proteoglycan 4
16	plasminogen	Ig gamma-3 chain C region	serine protease inhibitor	kallikrein B	serine protease inhibitor	Ig mu chain C region
17	coagulation factor XI	complement C3	Ig kappa chain C region	apolipoprotein A-I	complement factor H-related protein 1	selenoprotein P
18	isoform C of Fibulin-1	Ig alpha-1 chain C region	Ig gamma-3 chain C region	Ig gamma-3 chain C region	protease C1 inhibitor	serine protease inhibitor
19	apolipoprotein A-II	apolipoprotein A-II	Ig alpha-1 chain C region	Ig mu chain C region	complement C1q	Ig kappa chain C region
20	apolipoprotein C-I	vitronectin	complement C3	coagulation factor XI	Ig gamma-3 chain C region	complement factor H-related protein 1

**Figure 7:**
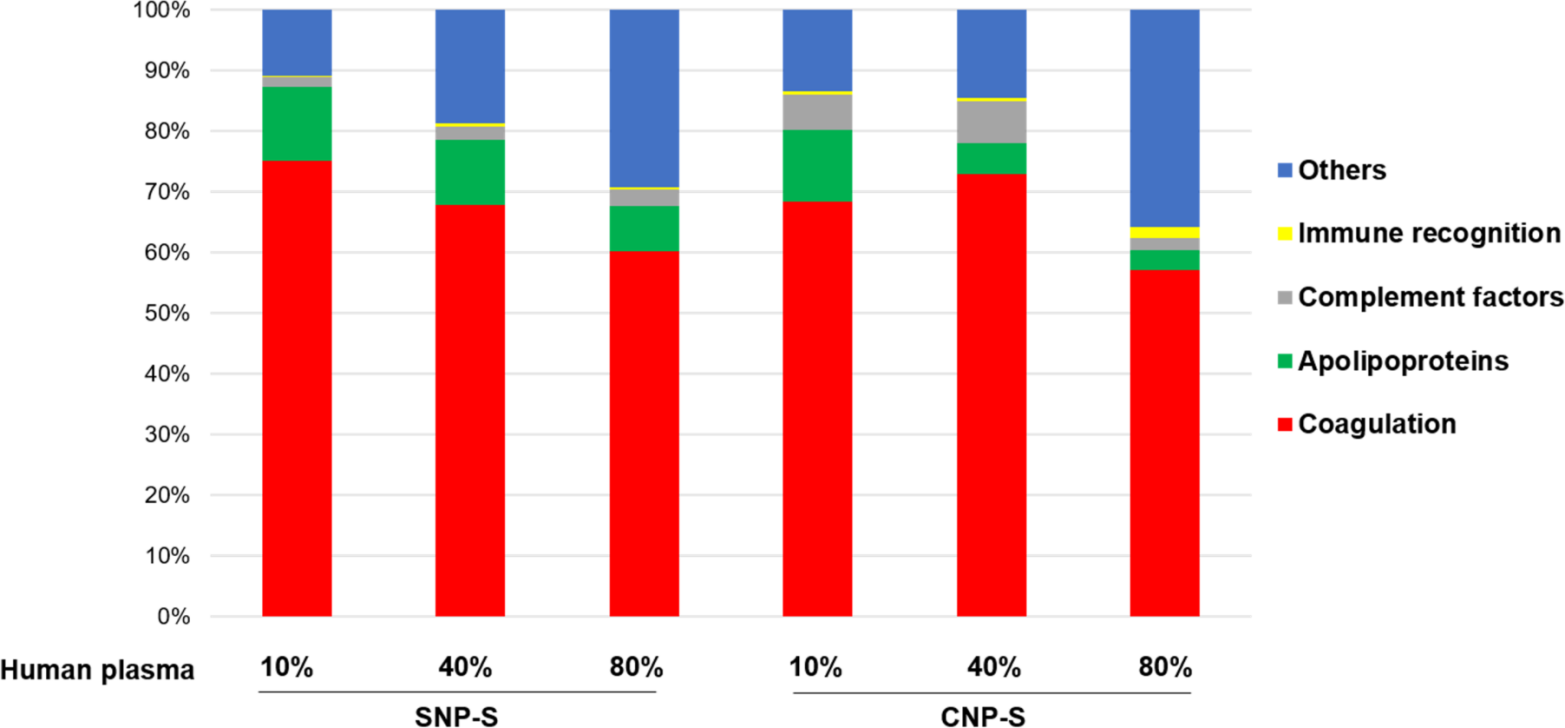
Classification of the human plasma corona proteins identified on small silica (SNP-S) and carbon (CNP-S) nanoparticles according to their biological functions. The LFQ intensity is used to calculate the percentages of protein groups.

Protein grouping (Figure 7) confirmed that the coagulation factors are highly enriched in the corona across all conditions although with different percentage (55–75%).

The presence of fibrinogen decreased significantly with increasing plasma concentration (80%) in silica corona, where it was displaced by less abundant proteins that had higher affinity proteins towards the NP surface, such as histidine-rich glycoprotein, kallikrein B and plasminogen as already shown in the literature [26].

Apolipoprotein A1, a major protein that forms the high-density lipoprotein (HDL), has shown to have a preferential affinity towards silica NPs since it was detected across all conditions. The findings were also in agreement with the SDS-PAGE results where a gel band of 28 kDa was detected only for silica NPs. Other HDL apolipopoproteins including apoA2 and A4 were also more abundant in the silica nanoparticle corona than the carbon one.

In contrast to what was observed for silica, fibrinogen was found to strongly bind to the CNPs also at higher concentrations of plasma. Similarly, ApoB100 and histidine-rich glycoprotein were enriched at 10% plasma, but they were displaced by other proteins such as vitronectin and ITIH4 at higher concentrations of plasma. Interestingly, albumin (66 kDa), the most abundant protein in human plasma, is outside the top 10 proteins identified with MS in all samples, confirming that the composition of the protein corona is independent of the protein original abundance.

In terms of molecular weights, most proteins found in the corona of both nanomaterials are between 20–60 kDa in weight, which accounts for about 70% of proteins (Supporting Information File 1, Figure S3). Around 8% of the total corona proteins have high molecular weights (>150 kDa).

### Effect of hard corona on agglomeration

**Figure 8:**
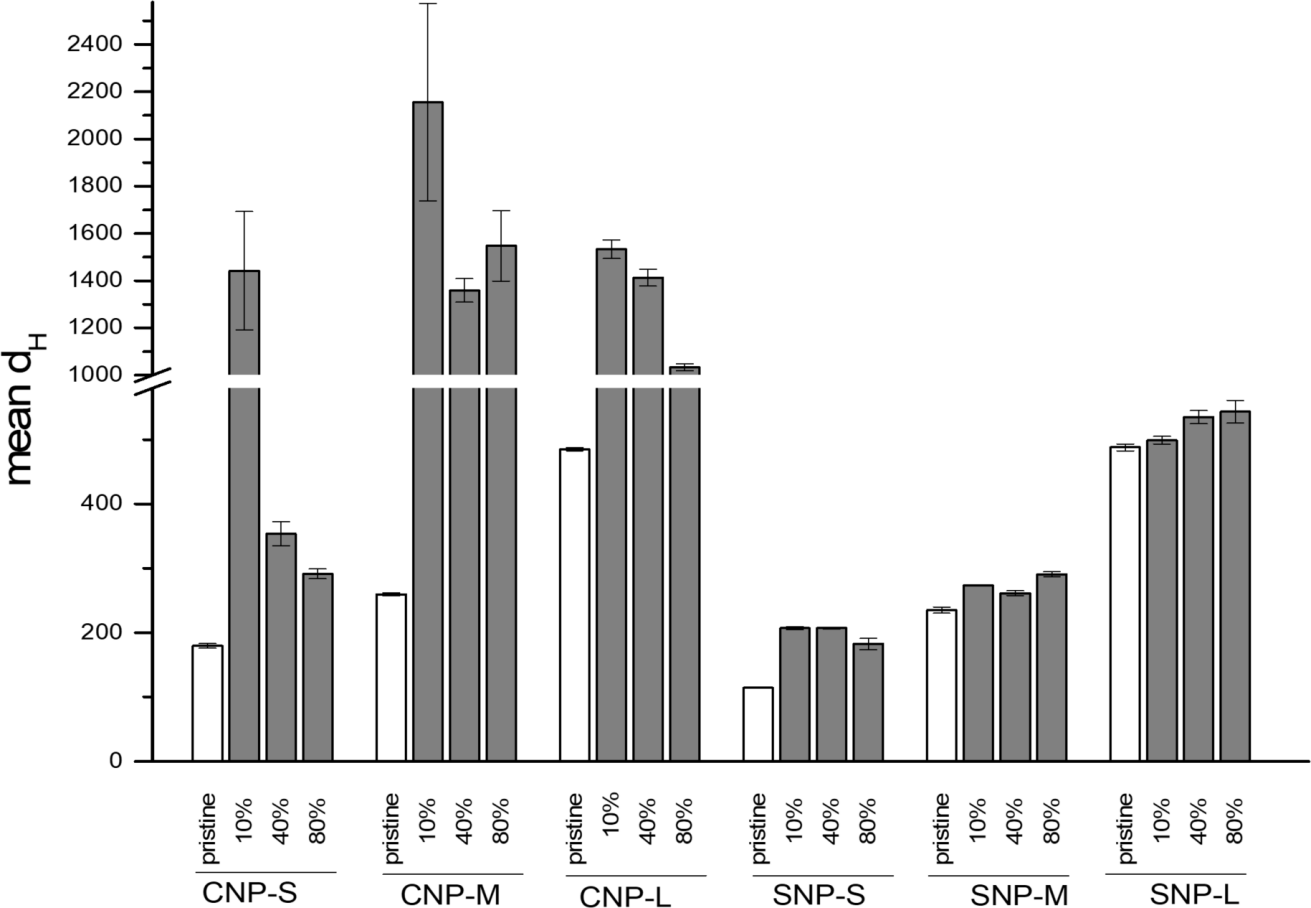
Effect of hard corona formed at different plasma concentrations on nanoparticles agglomeration in water.

The effect of the protein corona on the tendency of NPs to agglomerate was evaluated. The NP-hard corona complexes were diluted in PBS immediately after the sample preparation and the size distribution was measured by NTA. The mean hydrodynamic diameter of the particles with the protein corona generated at three different plasma concentrations (10, 40 and 80%) is compared in Figure 8 to the mean hydrodynamic diameter of the pristine NPs.

The presence of the hard protein corona induced substantial agglomeration in all CNPs and this effect was particularly enhanced for the protein corona formed in plasma at 10% concentration. By increasing the plasma concentration, the mean hydrodynamic size of the small carbon nanoparticles becomes similar to the NPs without the protein corona. Conversely, large and average size carbon nanoparticles remain highly agglomerated with a mean diameter greater than 1 µm. In the case of silica nanoparticles, no agglomeration was observed for all concentrations of plasma tested.

### Platelet aggregation

The effect of the NPs on platelet aggregation was measured using LTA. The NPs were added to platelet-rich plasma (PRP) in the absence or presence of two platelet activators, collagen and ADP.

**Figure 9:**
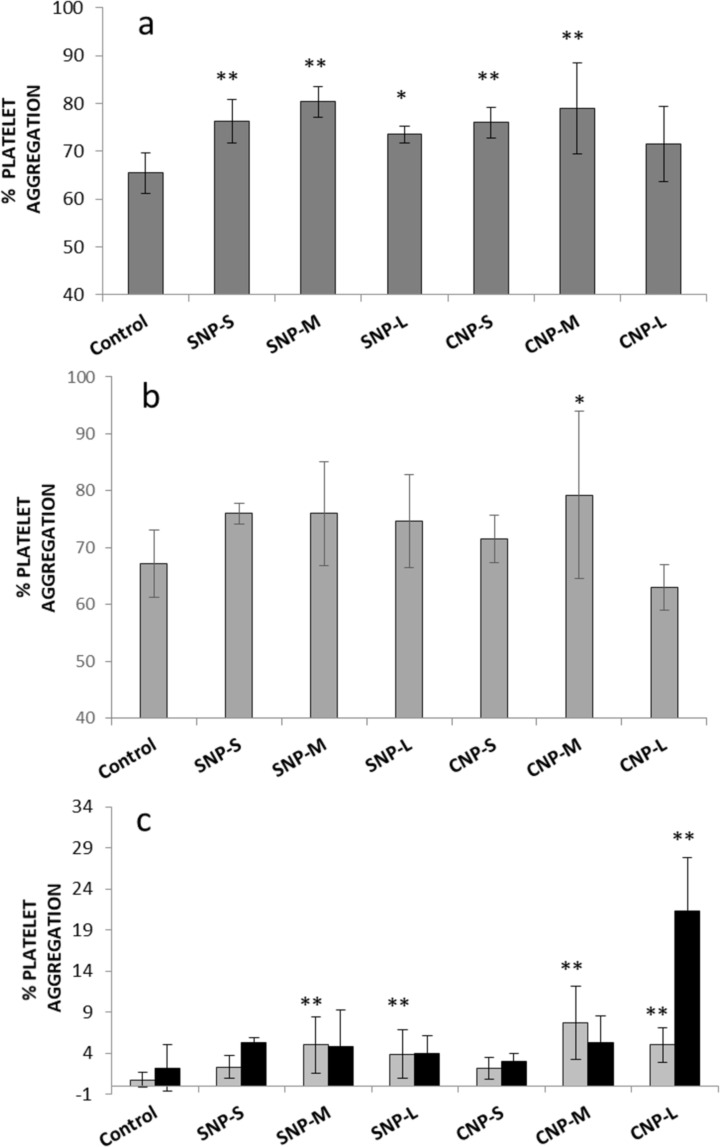
Effect of nanoparticles on platelet aggregation. a) Platelets activated by collagen; b) platelets activated by ADP; c) nonactivated platelets; after 5 (grey bars) and 40 (dark bars) minutes of incubation. ** *p* < 0.01, * *p* < 0.05.

In Figure 9a,b, we show the percent of platelet aggregation induced by silica and carbon NP samples after 5 min from the addition of ADP or collagen, which are known agonists for platelet aggregation. When the platelets were activated by collagen, a slight but significant increase in aggregation was observed for all samples. A similar trend was also observed in the presence of ADP as activator, albeit the values did not significantly differ from the control. In the case of medium and large CNPs (CNP-M and CNP-L), a progressive decrease in optical density was observed upon the addition of the NPs (Supporting Information File 1, Figure S4). To monitor this process, the optical density was measured up to 40 min after addition of the NPs in the absence of the activators (Figure 9c, Supporting Information File 1, Figure S5).

Significant aggregation was detected for both silica and carbon medium and large nanoparticles, with a major effect observed for CNP-L. This effect was previously reported by Bihiari and co-workers for SWCNTs [11] and was attributed to the formation of nanoparticle–platelet aggregates. Note that for large and medium size carbon nanoparticles, black aggregates were clearly visible at the end of the experiments (Supporting Information File 1, Figure S6).

### Platelet activation

**Figure 10:**
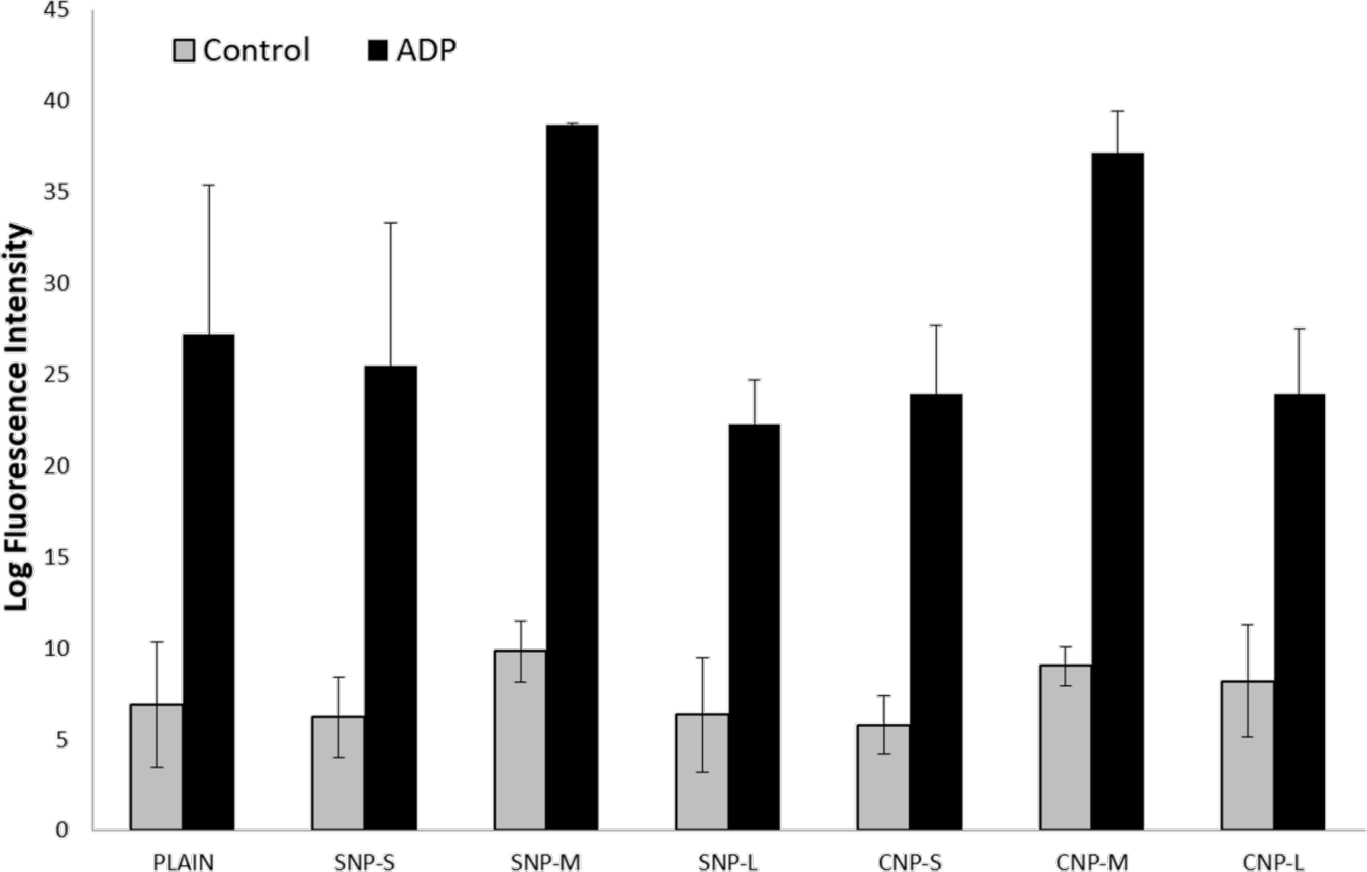
Effect of the nanoparticles on platelet activation measured as secretion of P-selectin.

The activation of platelets by the silica and carbon nanoparticles was evaluated by flow cytometry. Activation was evaluated by using a specific antibody, which binds the antigen CD62P (P-selectin) that is expressed on the surface of activated platelets (Figure 10). No significant activation was detected in the absence of activators. When platelets were activated with ADP, an increase of activation was observed for both silica and carbon nanoparticles of mean size only. This increase was evident, but not statistically significant due to the high variability of the response from one donor to the other.

The intensity of forward scattered (FS) and side scattered (SS) light was also measured to evaluate the size of platelets and the granularity, respectively (Supporting Information File 1, Figure S7). A slight increase of platelet size was observed in the presence of the silica and carbon NPs of medium size. The analysis of the platelet activation by flow cytometry is particularly critical in the presence of particles due to the possible interference in the intensity of scattered light. However, here this is not the case, since particles are clearly visible in the forward scattered (FS) and side scattered (SS) light plot (Supporting Information File 1, Figure S8) as separate populations having a size smaller than platelets, and therefore excluded by the measurement. However, possible interference may derive from aggregates of particles.

### Platelet adhesion

**Figure 11:**
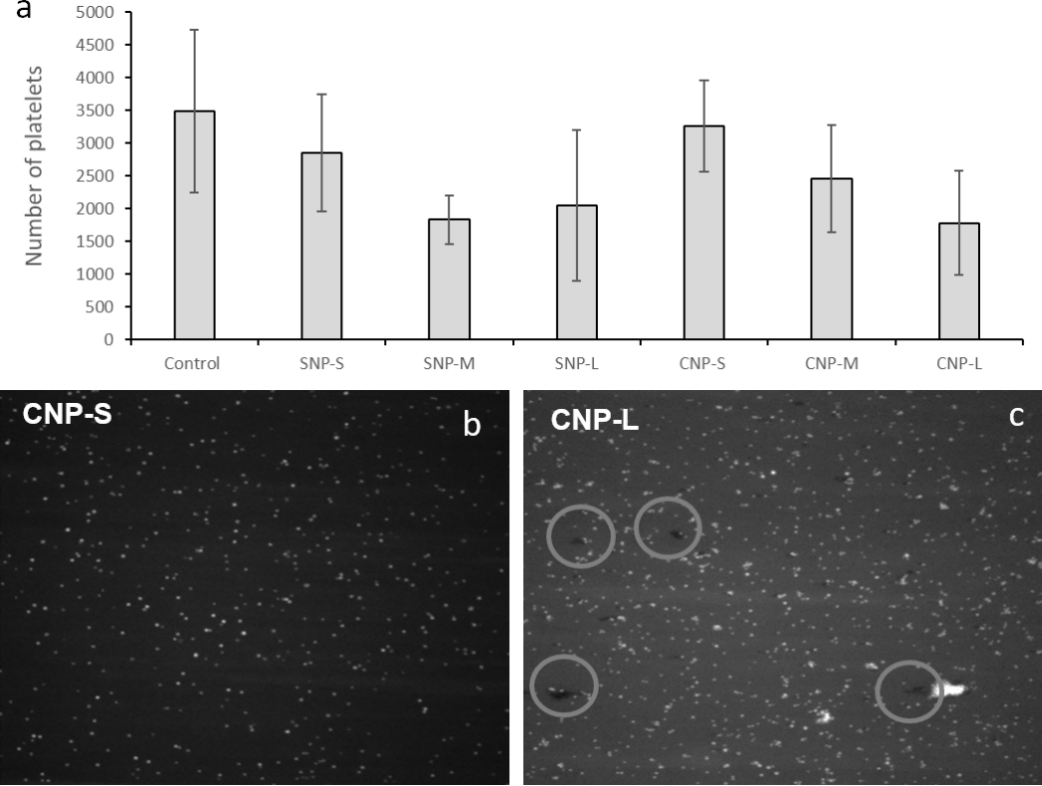
Effect of the nanoparticles on platelet adhesion. a) Total number of platelets adhering to the substrate; b) and c) representative images of the substrate during the measurements for CNP-S and CNP-L, respectively.

Activated platelets are physiologically programmed to adhere to the endothelial wall of damaged blood vessels. The VWF anchored to damaged endothelial cells plays a major role in this process, encouraging platelets to tether, roll and finally adhere at the site of damage. Dynamic platelet function assay (DPFA) was then used to investigate possible interference of the NPs on this process. This well-characterised assay monitors shear-mediated dynamic platelet interactions with surface-immobilised VWF. Adhesion was measured as the total number of platelets adhering to the substrate (Figure 11) in the presence or absence of the NPs.

A size dependent decrease in the number of platelets adhering to the substrate was detected (Figure 11a). Figure 11b shows two representative images of the VWF-coated microfluidic channel captured during the flow run. For CNP-L, large aggregates were observed at the surface of the substrate (circles). These aggregates were not visible for the other samples.

## Discussion

The identification of the correlations existing among the physical and chemical properties of a substance and the biological effects is a laborious but necessary process, allowing the design of more efficacious and safer medicines. In the case of (nano)biomaterials, this process is more challenging than for molecular substances, due to the higher number of parameters to be controlled. A library of nanomaterials that differs by one single property at time and accurate testing strategies are necessary. This is not always straightforward due to the interdependence between the various chemical and physical properties.

**Figure 12:**
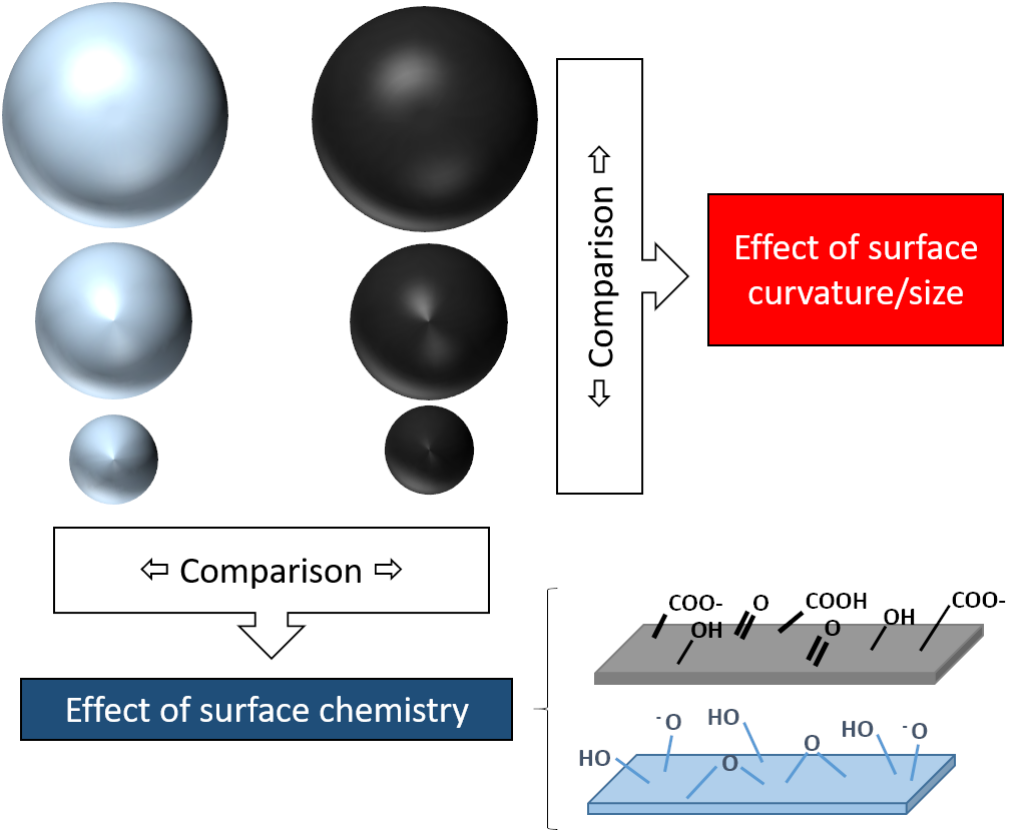
The strategy used to unravel possible structure–activity relationships in the present study.

In the present study, two sets of nanoparticles were prepared with the aim to specifically investigate the effect of the surface curvature and surface chemistry on platelet-dependent and independent aggregation, platelet activation and adhesion. Silica and carbon nanoparticles were chosen since both are highly studied for medical applications. Furthermore, being produced by wet methods, the selected nanoparticles have both hydrophilic surfaces and are negatively charged. Their comparison, therefore, excludes surface charge and hydrophilicity as variables to be investigated. In Figure 12 we summarise the strategy used to unravel possible SARs.

This strategy allowed us to identify the surface chemistry as the key factor in the protein corona composition while both surface chemistry and size modulate a platelet-independent aggregation potential of particles in blood.

Platelet aggregation is a complex process modulated by several chemical and physical parameters. Ordinarily platelets circulate in blood in a quiescent state near the endothelial cells lining the blood vessels without forming stable adhesions. After infringement of the vasculature proteins like VWF, collagen and fibronectin are exposed on the sub-endothelial matrix and act as ligands for the platelet surface receptors, such as glycoproteins like GPVI and GPIbα, that lead the platelet adhesion to the affected area [35–37]. These receptor–ligand interactions initiate a cascade of intracellular responses resulting in amplification of platelet activation through the secretion of soluble agonists including thromboxane A2 (TXA2) and ADP. TXA2 and ADP act jointly with the engaged platelet receptors to mobilize intracellular Ca^2+^, which instigates platelet shape change, degranulation, and up-regulation of the adhesive function of another platelet surface receptor, integrin αIIbβ3 [35]. The active conformation of αIIbβ3 integrin can then bind fibrinogen, VWF and fibronectin with high affinity, allowing haemostatic platelets to aggregate and thrombus formation [38]. Fibrinogen plays a key role in platelet aggregation, forming bridges between platelets and acting as an aggregation glue. On the other hand, fibrinogen also has a key role in NP aggregation. Fibrinogen has a high affinity for surfaces [39], and it is commonly present in the protein layer/corona of several materials [26,40]. On hydrophilic surfaces, this protein tends to be displaced by other proteins by a mechanism known as Vroman’s effect [39]. However, in some cases fibrinogen remains bound to the surface and undergoes conformational changes, thus exposing cryptic domains. Platelets may adhere to fibrinogen immobilised onto biomaterials through integrins, a mechanism that may lead to thrombotic events. Furthermore, in the case of NPs, this protein may act as glue in a similar way to that observed with platelets [21], inducing NP aggregation. However, in this case the effect is not due to the interaction with integrin, but it is a non-specific process due to the tendency of fibrinogen to form fibrils similar to fibrin. This tendency is a consequence of the specific fibrinogen arrangement onto surfaces, modulated by the surface properties [23].

Fibrinogen is not the only plasma protein that may be activated by the surface-inducing pro-thrombotic effects. Several studies report the activation by anionic NPs of the coagulation factor XII [17–18], an effect that is modulated by the NP size [41].

Fibrinogen was found in in both SNP-S and CNP-S protein corona, regardless of the NP size, but its presence was particularly enhanced in CNP-S when incubated with highly concentrated plasma. A similar behaviour was already reported for SNPs indicating that the surface area / protein abundance in the biological milieu strongly affects the protein binding to these surfaces [26].

Both SNPs and CNPs interact with plasma proteins forming a protein corona. The presence of the protein corona clearly induced platelet-independent agglomeration of carbon nanoparticles but not for silica (Figure 8). Notably, aggregation was observed for medium size CNPs and large CNP at all plasma concentrations tested, while in the case of small CNPs, aggregation was observed only with 10% of plasma, corresponding to the condition used in in vitro tests, but not in vivo. The latter is in agreement with that recently found on Au nanoparticles by Ho and co-workers [42].

The differences in platelet-independent aggregation behaviour observed among the SNP and CNP might be a consequence of the different composition of the protein corona, or to a different arrangement of protein molecules at the surface, as a consequence of a different ability of the surfaces to interact with the proteins [22]. The affinity of a protein for a certain surface and the mode of interaction rise from the interplay of electrostatic interactions, hydrogen bondings, and hydrophobic forces [32]. Both nanomaterials are negatively charged at physiological pH. However, SNPs exhibit a less negative zeta potential when compared to CNPs of a similar size, likely due to a lower mean Brönsted acidity of the CNPs surface, being the density of acidic carboxyl/phenolic groups at the carbon surface similar to the expected density of silanols (Si–OH) at a fully hydroxylated surface, i.e., of 4–5 groups/nm^2^ [31–32]. Both surfaces exhibit surface sites able to form hydrogen bonds or hydrophobic interaction with proteins. However, such tendency may be different since hydrogen bond formation obeys geometrical constraints due to the directional character of this bond. On the other hand, both silica and carbon surfaces exhibit hydrophobic patches, i.e., siloxane bridges and carbon–carbon bonds, respectively. These moieties have a different nature, exhibiting a higher dipolar character.

Previous studies reported fibrinogen-induced aggregation for silica nanoparticles [23,40]. However, in these studies, pyrogenic silica was used. This material is very different to silica produced by sol–gel methods, being formed by large aggregates and having a surface with a lower degree of hydrophilicity [20]. A different arrangement of the fibrinogen molecule at the surface of silica is therefore likely to occur. In fact, we previously reported that the tendency of fibrinogen to self-assemble to form fibrin-like fibrils increased by decreasing the hydrophilic character of silica [23].

When incubated in the presence of platelets, SNPs induced only mild aggregation (Figure 9). This is in agreement with that previously found on SNPs of similar size [16]. Similarly, CNPs induce mild aggregation only, regardless of the presence of several proteins involved in the coagulation cascade in the hard corona of the NPs. On the other hand, with large carbon nanoparticles, aggregation was observed for a prolonged incubation time. This process does not involve platelet activation and appears related to the ability of particles to act as bridges among platelets, similar to that observed by other authors with other carbon nanomaterials [11,13]. This was confirmed for large nanoparticles (Figure 10), while for medium size particles, platelet activation cannot be excluded.

In light of this evidence, the observed reduction of VWF-mediated adhesion of platelets to the endothelial wall induced by all NPs should be regarded as a consequence of the sequestration of platelets by particles. In fact, this effect is more evident for large particles.

The different aggregation potential of CNPs depending on their size may explain the contrasting data found in the literature on isometric carbon nanoparticles. In fact, secretion of P-selectin in vitro was observed for carbon black [13] but not diesel exhaust particles [11], while platelet aggregation was observed for amorphous CNPs but not for the small-sized fullerenes [10]. Note however that limited information relating to the physicochemical properties of the materials was given in these studies, making a critical analysis of the results difficult. Moreover, while CNTs were reported to induce platelet aggregation [10–11] CNPs did not. This supports the hypothesis by De Paoli Lacerda and co-workers that an elongated shape is necessary for this process [12].

## Conclusion

In conclusion, the present study suggests that highly stable and monodispersed NPs may generate aggregates in specific exposing conditions by platelet-independent pathways and stresses the importance of the need to characterise nanomaterials in relevant biological fluids (in this case blood plasma or blood). This result should be regarded with concern, since aggregates might induce vessel occlusion in vivo. However, the reduction of the diameter to less than 100 nm appears to improve the stability of CNPs and possibly their biocompatibility. Further in vivo investigations will be necessary to confirm this hypothesis.

## Supporting Information

File 1 Additional figures.

## References

[1] Coty J-B, Vauthier C (2018). J Controlled Release.

[2] Khorasani A A, Weaver J L, Salvador-Morales C (2014). Int J Nanomed.

[3] Fadeel B (2013). J Intern Med.

[4] Ilinskaya A N, Dobrovolskaia M A (2013). Nanomedicine (London, U K).

[5] Deng Z J, Liang M T, Monteiro M, Toth I, Minchin R F (2011). Nat Nanotechnol.

[6] Kushida T, Saha K, Subramani C, Nandwana V, Rotello V M (2014). Nanoscale.

[7] Dobrovolskaia M A, Patri A K, Simak J, Hall J B, Semberova J, De Paoli Lacerda S H, McNeil S E (2012). Mol Pharmaceutics.

[8] Jones C F, Campbell R A, Franks Z, Gibson C C, Thiagarajan G, Vieira-de-Abreu A, Sukavaneshvar S, Mohammad S F, Li D Y, Ghandehari H (2012). Mol Pharmaceutics.

[9] Gubala V, Giovannini G, Kunc F, Monopoli M P, Moore C J (2020). Cancer Nanotechnol.

[10] Semberova J, De Paoli Lacerda S H, Simakova O, Holada K, Gelderman M P, Simak J (2009). Nano Lett.

[11] Bihari P, Holzer M, Praetner M, Fent J, Lerchenberger M, Reichel C A, Rehberg M, Lakatos S, Krombach F (2010). Toxicology.

[12] De Paoli Lacerda S H, Semberova J, Holada K, Simakova O, Hudson S D, Simak J (2011). ACS Nano.

[13] Holzer M, Bihari P, Praetner M, Uhl B, Reichel C, Fent J, Vippola M, Lakatos S, Krombach F (2014). J Appl Toxicol.

[14] Khandoga A, Stoeger T, Khandoga A G, Bihari P, Karg E, Ettehadieh D, Lakatos S, Fent J, Schulz H, Krombach F (2010). J Thromb Haemostasis.

[15] Khandoga A, Stampfl A, Takenaka S, Schulz H, Radykewicz R, Kreyling W, Krombach F (2004). Circulation.

[16] Corbalan J J, Medina C, Jacoby A, Malinski T, Radomski M W (2012). Int J Nanomed.

[17] Jiang L, Li Y, Li Y, Guo C, Yu Y, Zou Y, Yang Y, Yu Y, Duan J, Geng W (2015). Toxicol Res (Cambridge, U K).

[18] Yoshida T, Yoshioka Y, Morishita Y, Aoyama M, Tochigi S, Hirai T, Tanaka K, Nagano K, Kamada H, Tsunoda S (2015). Nanotechnology.

[19] Napierska D, Thomassen L C J, Rabolli V, Lison D, Gonzalez L, Kirsch-Volders M, Martens J A, Hoet P H (2009). Small.

[20] Gazzano E, Ghiazza M, Polimeni M, Bolis V, Fenoglio I, Attanasio A, Mazzucco G, Fubini B, Ghigo G (2012). Toxicol Sci.

[21] Marucco A, Gazzano E, Ghigo D, Enrico E, Fenoglio I (2014). Nanotoxicology.

[22] Fenoglio I, Fubini B, Ghibaudi E M, Turci F (2011). Adv Drug Delivery Rev.

[23] Marucco A, Turci F, O’Neill L, Byrne H J, Fubini B, Fenoglio I (2014). J Colloid Interface Sci.

[24] Kokalari I, Gassino R, Giovannozzi A M, Croin L, Gazzano E, Bergamaschi E, Rossi A M, Perrone G, Riganti C, Ponti J (2019). Free Radical Biol Med.

[25] Stöber W, Fink A, Bohn E (1968). J Colloid Interface Sci.

[26] Monopoli M P, Walczyk D, Campbell A, Elia G, Lynch I, Baldelli Bombelli F, Dawson K A (2011). J Am Chem Soc.

[27] Ralph A, Somers M, Cowman J, Voisin B, Hogan E, Dunne H, Dunne E, Byrne B, Kent N, Ricco A J (2016). Cardiovasc Eng Tech.

[28] Cowman J, Richter L, Walsh R, Keegan N, Tinago W, Ricco A J, Hennessy B T, Kenny D, Dunne E (2019). Platelets.

[29] Dunne E, Qi Q M, Shaqfeh E S, O’Sullivan J M, Schoen I, Ricco A J, O’Donnell J S, Kenny D (2019). Blood.

[30] Pietroiusti A, Massimiani M, Fenoglio I, Colonna M, Valentini F, Palleschi G, Camaioni A, Magrini A, Siracusa G, Bergamaschi A (2011). ACS Nano.

[31] Iler R K (1979). The Chemistry of Silica.

[32] Rimola A, Costa D, Sodupe M, Lambert J-F, Ugliengo P (2013). Chem Rev.

[33] Ek S, Root A, Peussa M, Niinisto L (2001). Thermochim Acta.

[34] Lundqvist M, Augustsson C, Lilja M, Lundkvist K, Dahlbäck B, Linse S, Cedervall T (2017). PLoS One.

[35] McFadyen J D, Kaplan Z S (2015). Transfus Med Rev.

[36] Ruggeri Z M (2009). Microcirculation.

[37] Jackson S P (2007). Blood.

[38] McFadyen J D, Jackson S P (2013). Thromb Haemostasis.

[39] Vroman L, Adams A L, Fischer G C, Munoz P C (1980). Blood.

[40] Kendall M, Ding P, Kendall K (2011). Nanotoxicology.

[41] Hao F, Liu Q S, Chen X, Zhao X, Zhou Q, Liao C, Jiang G (2019). ACS Nano.

[42] Ho Y T, Azman N‘A, Loh F W Y, Ong G K T, Engudar G, Kriz S A, Kah J C Y (2018). Bioconjugate Chem.

