# Supplementary material for: Identification of physicochemical properties that modulate nanoparticle aggregation in blood
Source: Beilstein J Nanotechnol. 2020 Apr 3;11:550–67. doi: 10.3762/bjnano.11.44 (PMC7136551; doi:10.3762/bjnano.11.44)
Supplement: File 1 — Additional figures. [file Beilstein_J_Nanotechnol-11-550-s001.pdf]

BEILSTEIN JOURNAL OF NANOTECHNOLOGY

Supporting Information

for

Identification of physicochemical properties that modulate nanoparticle aggregation in blood

Ludovica Soddu, Duong N. Trinh, Eimear Dunne, Dermot Kenny, Giorgia Bernardini, Ida Kokalari, Arianna Marucco, Marco P. Monopoli and Ivana Fenoglio

Beilstein J. Nanotechnol. **2020,** *11,* 550–567. doi:10.3762/bjnano.11.44

Additional figures

License and Terms: This is a supporting information file under the terms of the Creative Commons Attribution License (http://creativecommons.org/licenses/by/4.0). Please note that the reuse, redistribution and reproduction in particular requires that the authors and source are credited.
The license is subject to the *Beilstein Journal of Nanotechnology* terms and conditions: (https://www.beilstein-journals.org/bjnano)

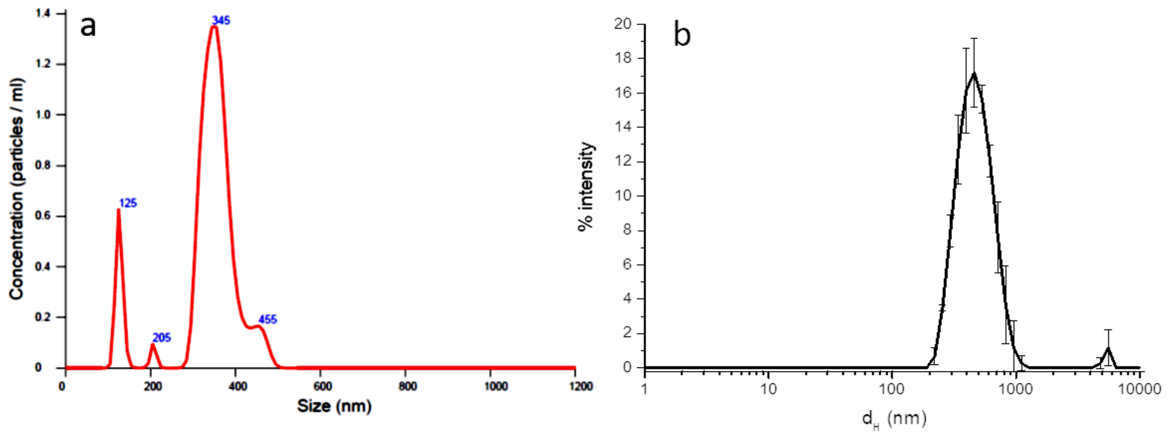

Figure S1: Representative size distribution measured by a) NTA and b) DLS of the sample SNP-L.

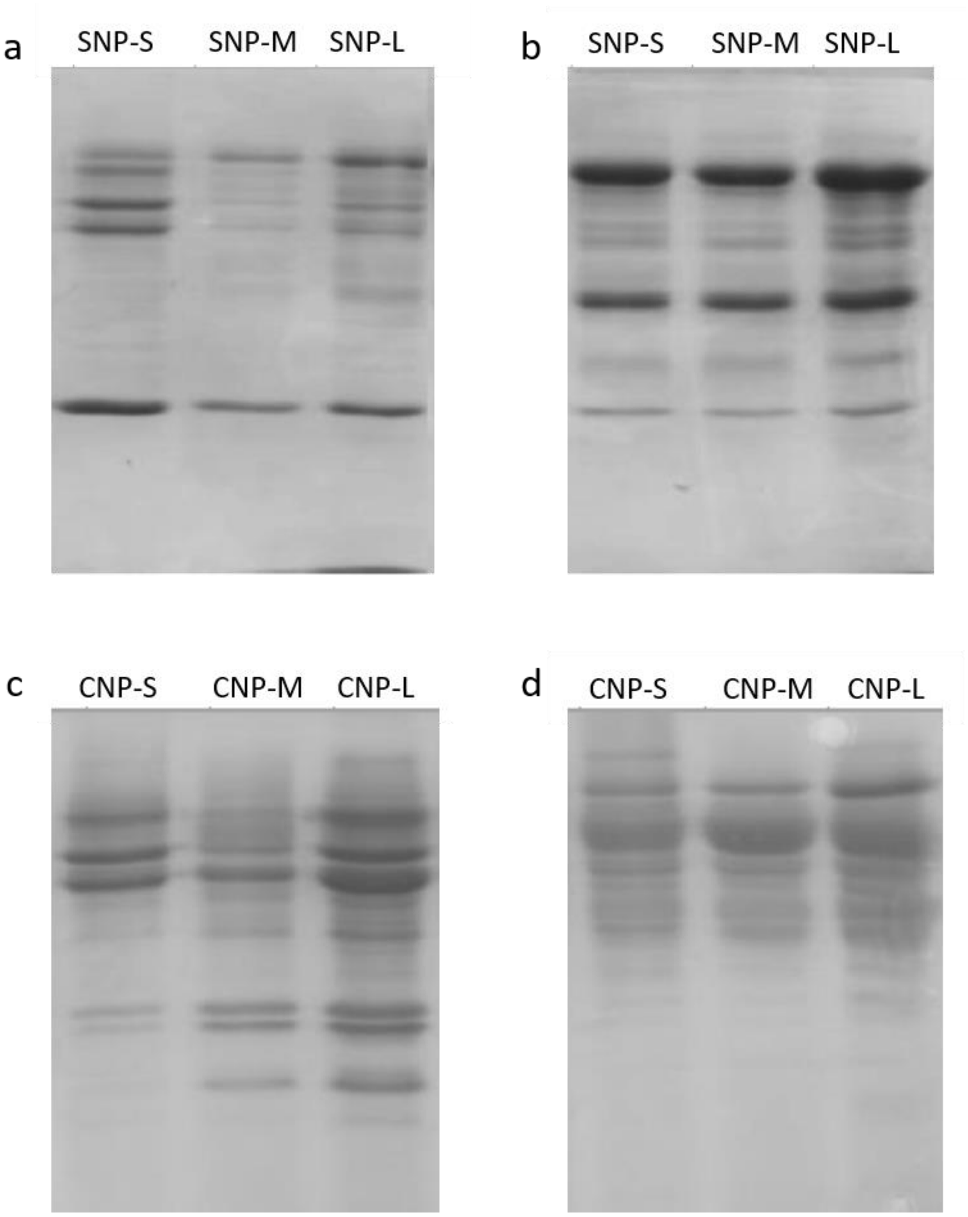

Figure S2: Effect of size on the composition of the protein corona. Panel a) and c) 10% human plasma, b) and d) 80% human plasma.

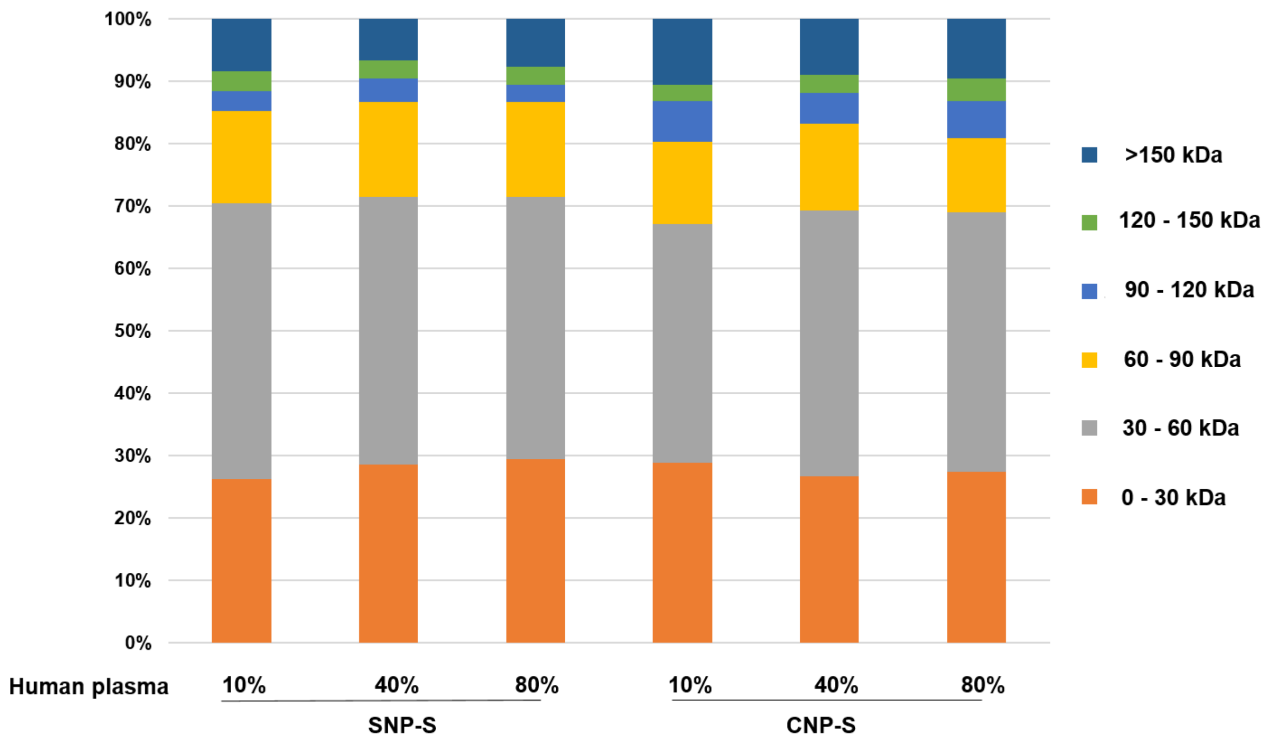

Figure S3: Classification of the human plasma corona proteins identified on small silica and carbon NPs by LC-MS according to the molecular weight (MW) at three plasma concentration.

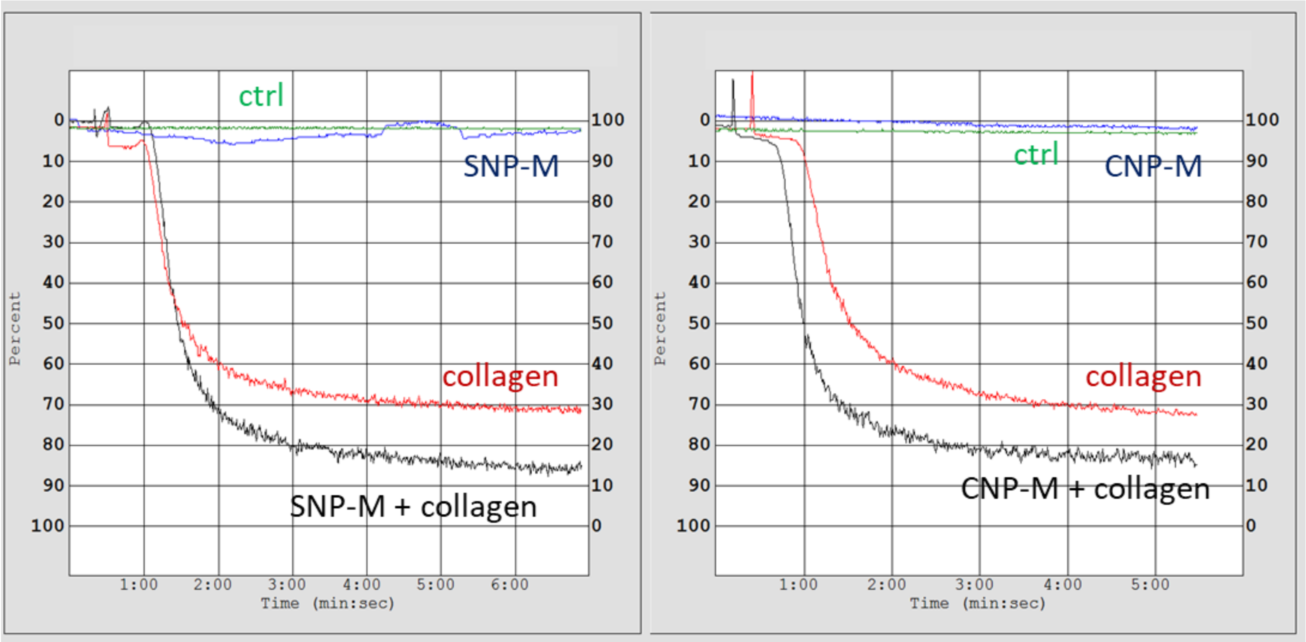

Figure S4: Representative plots of platelet aggregation. Effect of SNP-M and CNP-M on aggregation of platelets activated with collagen.

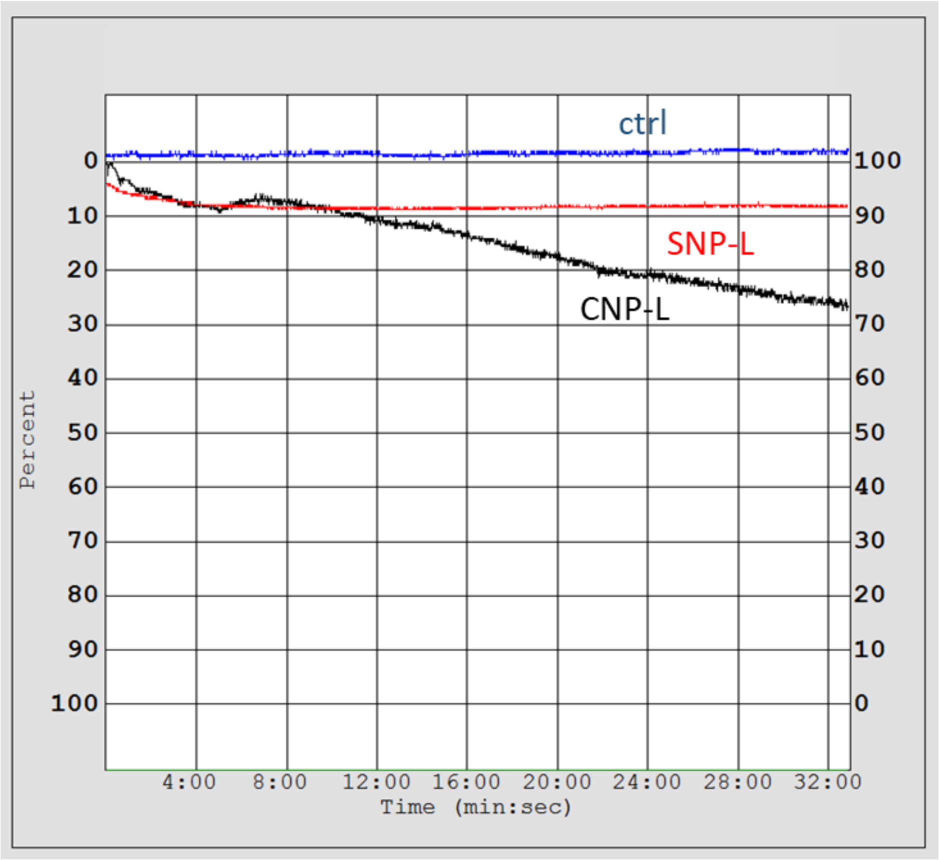

Figure S5: Effect of SNP-L and CNP-L on platelet aggregation in the absence of activators.

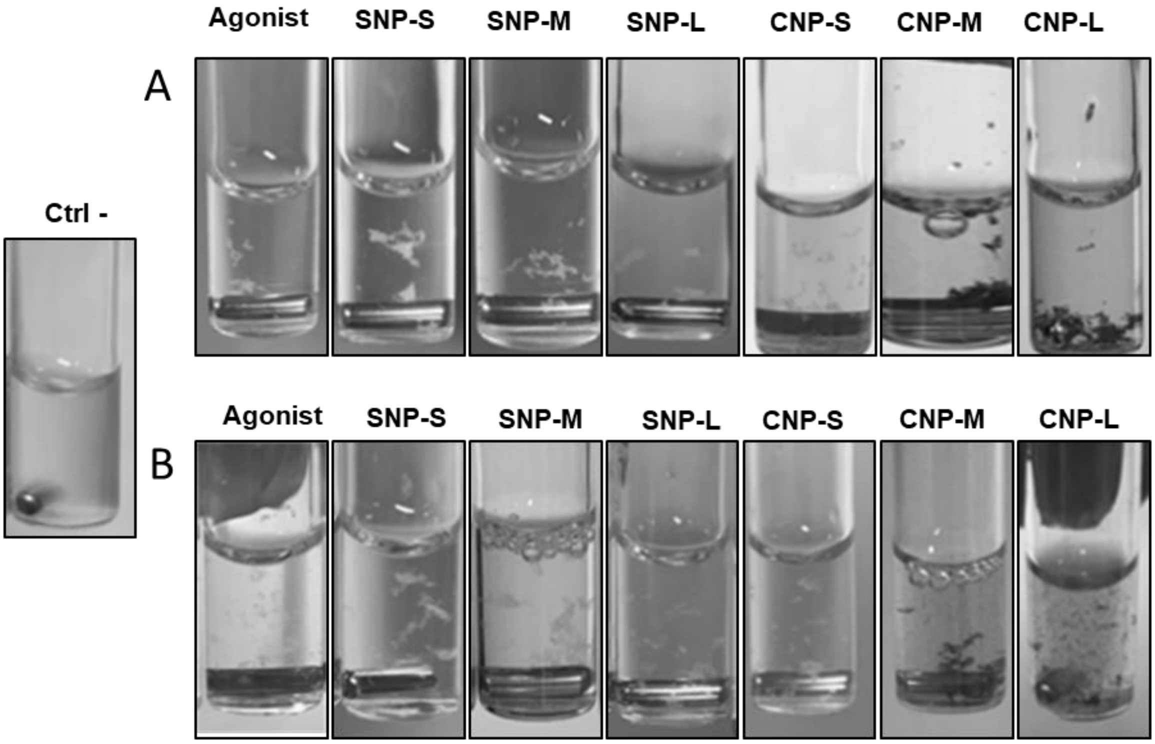

Figure S6: Images of PRP after addition of the activator (A. collagen; B. ADP) and the nanoparticles. Ctrl- : PRP.

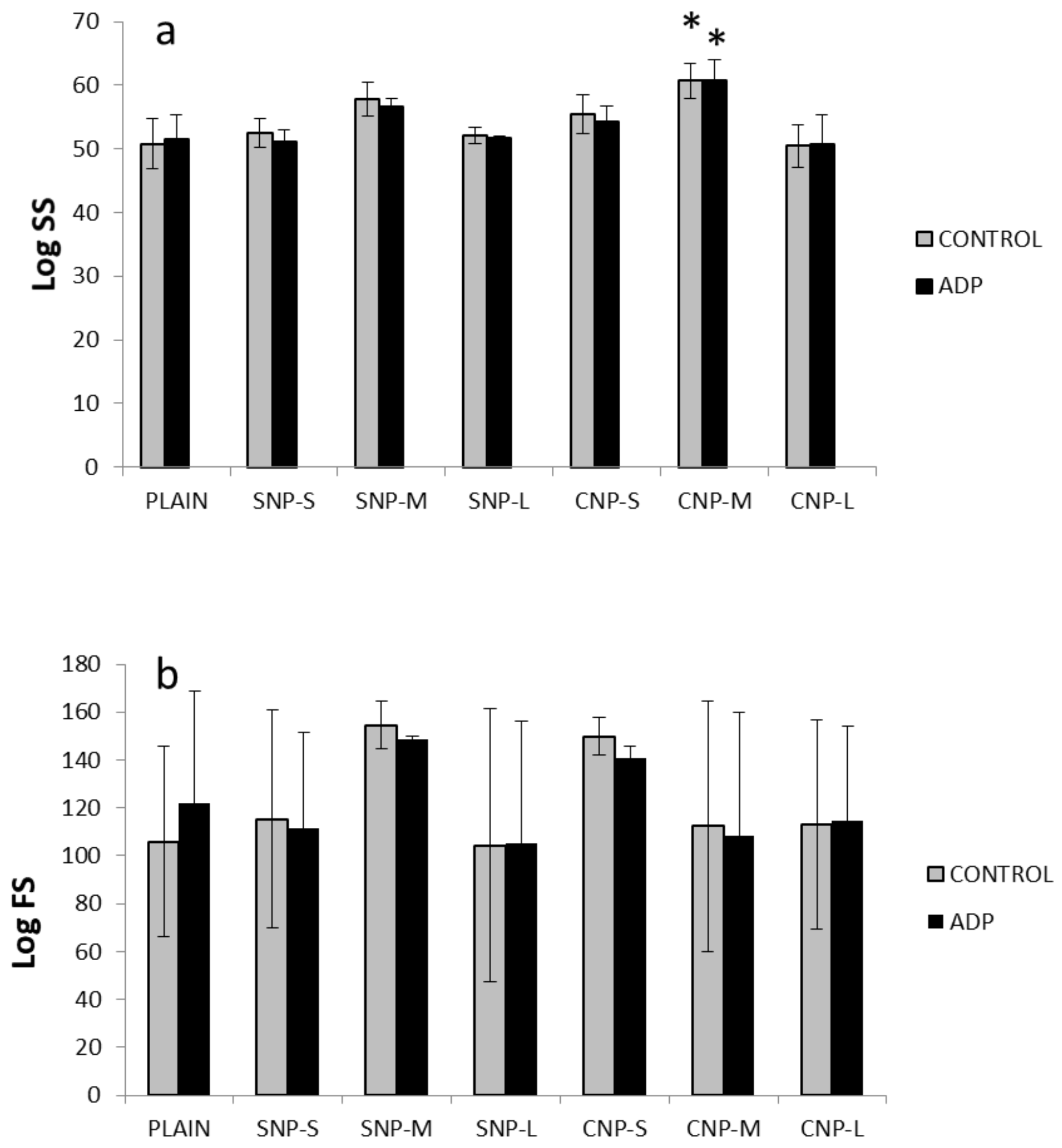

Figure S7: Effect of the nanoparticles on size and granularity of platelets evaluated by flow cytometry. a) Intensity of forward (FS) and b) side (SS) scattered light. * $p < 0.05$.

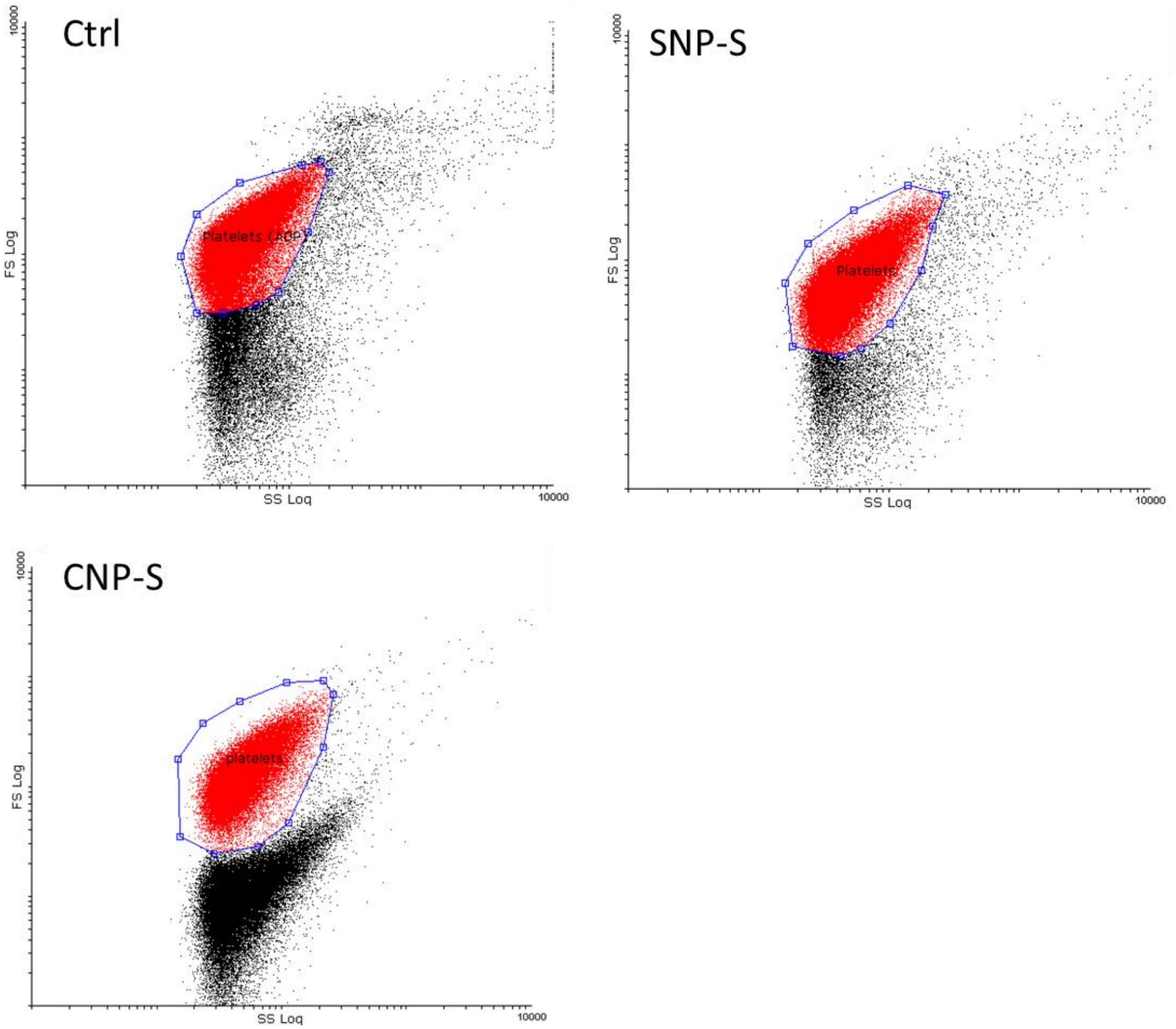

Figure S8: Representative FS/SS plot.
